# *Fusobacterium nucleatum* infection modulates the transcriptome and epigenome of HCT116 colorectal cancer cells in an oxygen-dependent manner

**DOI:** 10.1038/s42003-024-06201-w

**Published:** 2024-05-08

**Authors:** Barath Udayasuryan, Zirui Zhou, Raffae N. Ahmad, Polina Sobol, Chengyu Deng, Tam T. D. Nguyen, Shivanie Kodikalla, Ryan Morrison, Ishan Goswami, Daniel J. Slade, Scott S. Verbridge, Chang Lu

**Affiliations:** 1 School of Biomedical Engineering and Sciences, Virginia Tech-Wake Forest University, Blacksburg, VA USA; 2 Department of Chemical Engineering, Virginia Tech, Blacksburg, VA USA; 3 Department of Biochemistry, Virginia Tech, Blacksburg, VA USA

**Keywords:** Epigenomics, Cancer genomics

## Abstract

*Fusobacterium nucleatum*, a gram-negative oral bacterium, has been consistently validated as a strong contributor to the progression of several types of cancer, including colorectal (CRC) and pancreatic cancer. While previous in vitro studies have shown that intracellular *F. nucleatum* enhances malignant phenotypes such as cell migration, the dependence of this regulation on features of the tumor microenvironment (TME) such as oxygen levels are wholly uncharacterized. Here we examine the influence of hypoxia in facilitating *F. nucleatum* invasion and its effects on host responses focusing on changes in the global epigenome and transcriptome. Using a multiomic approach, we analyze epigenomic alterations of H3K27ac and global transcriptomic alterations sustained within a hypoxia and normoxia conditioned CRC cell line HCT116 at 24 h following initial infection with *F. nucleatum*. Our findings reveal that intracellular *F. nucleatum* activates signaling pathways and biological processes in host cells similar to those induced upon hypoxia conditioning in the absence of infection. Furthermore, we show that a hypoxic TME favors *F. nucleatum* invasion and persistence and therefore infection under hypoxia may amplify malignant transformation by exacerbating the effects induced by hypoxia alone. These results motivate future studies to investigate host-microbe interactions in tumor tissue relevant conditions that more accurately define parameters for targeted cancer therapies.

## Abstract (web summary)

Multiomic study reveals that tumor microenvironmental factors such as hypoxia influence intracellular infection of *Fusobacterium nucleatum* in colorectal cancer cells and provides clues to malignant transformation of infected cells.

## Introduction

Tumor-associated bacteria, now considered a hallmark of cancer^1^, can impact tumor progression and response to therapy^2^. Systematic characterizations of the tumor microbiome in several tumor types have revealed diverse intracellular bacteria within tumors^3,4^. Far from a random infection of the tumor site, these microbes form highly organized microniches within the tumor and can modulate myriad tumor microenvironmental (TME) factors^5^. Furthermore, studies have shown that intracellular bacteria directly promote metastatic colonization in mouse models of breast cancer^6^. Evolutionarily, invasive bacterial species exhibit adaptations that facilitate their survival, immune evasion, and dissemination. However, there is still limited knowledge on the strategies bacteria adopt for intracellular survival in cancer cells, their long-term epigenetic imprints on the host cells, and if or how their elimination can limit tumor progression^7^.

Normally found within the oral cavity, *Fusobacterium nucleatum* is a commensal, Gram-negative, anaerobic bacilli that acts as a bridge species within the oral cavity and facilitates biofilm formation^8^. However, it behaves as an opportunistic pathogen in disease conditions including gingivitis, periodontitis, inflammatory bowel disease, myocarditis, and in preterm birth complications^9,10^. It is believed to primarily disseminate from the mouth through the bloodstream to facilitate extra-oral colonization and infection^11^.

More than a decade ago, *F. nucleatum* was found to be selectively enriched in CRC tumors in comparison to adjacent healthy tissue^12,13^. Subsequent clinical studies have further corroborated its role as a potential ‘oncomicrobe’, providing evidence that patients harboring *F. nucleatum* within their tumors exhibit increased mortality, resistance to chemotherapy, and greater risk of relapse after chemotherapy or tumor resection^14–17^. The host cell’s response to *F. nucleatum* infection can be multifactorial. We have previously shown that *F. nucleatum* binding and invasion of host cells induces the robust secretion of select cytokines that can impact tumor cell proliferation and migration even in the absence of immune cell involvement^18,19^. Furthermore, *F. nucleatum* can persist within the primary tumor cell for a prolonged period and even travel within the tumor cell to distant metastatic sites^20^. Concurrently, it has been shown that ICAM-1 overexpression in *F. nucleatum* infected CRC cells can facilitate endothelial adhesion and extravasation to promote metastatic spread^21^. Other microbial species co-localizing within the tumor microbiome can degrade chemotherapeutics such as 5-fluorouracil to enable *F. nucleatum* persistence^22^. Taken together, these studies suggest that *F. nucleatum* infection in tumors drives the formation of malignant phenotypes of tumor cells. However, differences in human and murine biology have impeded the establishment of a stably colonized murine model to study *F. nucleatum* pathogenicity^23^. An overarching question in the field is what are the factors within a permissive TME that convert symbionts such as *F. nucleatum* into oncomicrobes? Current findings have only begun to reveal features of this host-pathogen interaction and there remains much to be understood on how *F. nucleatum* adapts to the TME, modulates host cell response, and promotes malignant transformation of the host cells, including the effect of sustained epigenetic imprints on infected cancer or even normal cells.

In vitro experiments have usually been designed for the study of *F. nucleatum* infection of cell lines in normoxic conditions (18–21% oxygen). However, physiological oxygen conditions, i.e. physoxia (2–6%) and hypoxic ( < 1%) conditions more accurately recapitulate the TME^24^. Measurements of oxygen levels using positron emission tomography (PET) or biomarkers such as HIF-1ɑ indicate the prevalence of hypoxia in CRC^25^. However, the localized oxygen tension within infected microniches in vivo is less clear due to practical challenges in direct and real-time oxygen detection. As tumors grow they develop a hypoxic core due to rapid proliferation and limited vascularization. In addition, inflammation-induced stromal and immune infiltration of the TME can increase cellular oxygen demand to result in local oxygen deficits. Hypoxia in CRC potentiates the development of more aggressive tumor cell phenotypes by altering signaling pathways, modulating cytoskeletal rearrangements, promoting epithelial-mesenchymal transition (EMT), reshaping metabolism, inducing angiogenesis, and exhibiting increased drug resistance^26^. Moreover, these features favor microbial intracellular invasion and persistence. Low oxygen can trigger variations in metabolite production, promote biofilm formation, and attenuate host defenses to impact host-microbe interactions, as in the case of *Pseudomonas aeruginosa*, *Staphylococcus aureus*, and *Staphylococcus epidermidis* in cystic fibrosis^27^. In addition, anaerobic microenvironments have been shown to promote infection of *Shigella flexneri* into the gut epithelium by activating virulence mechanisms^28^. Since *F. nucleatum* is an anaerobic organism, we hypothesized that hypoxic environments would be conducive to its growth and its virulence in hypoxic cells would vary from its impact on normoxic cells. Fusobacterial localization to hypoxic environments could also help to explain spatial patterns of tumor colonization observed in vivo within poorly vascularized tumor niches^5^. In vitro modeling of infection in CRC derived spheroids revealed *F. nucleatum* proliferation was significantly increased in large spheroids compared to smaller spheroids, suggesting a key role of a hypoxic core to promote bacterial viability^29^. Hypoxia extensively modifies the epigenetic and transcriptomic landscape of cells^30^, and hence, global profiling of alterations induced by hypoxia may reveal clues as to how hypoxia modulates *F. nucleatum* persistence and pathogenicity.

Prior studies have revealed epigenomic and/or transcriptomic alterations induced by *F. nucleatum* in normoxia on non-cancerous cell lines including primary human colonic epithelial cells, human carotid artery endothelial cells^31^, human gingival fibroblasts^32,33^, rat osteoblasts^34^, and human immortalized oral epithelial cells^35^. Microarray data analysis of *F. nucleatum* infection of Caco-2 CRC cells, identified a role for CEP5 centrosomal protein that can impact cell cycle and apoptosis^36^. Other studies have focused on the RNA landscape of multiple *F. nucleatum* strains^37^ or alterations in gene expression within *F. nucleatum* upon infection of Caco-2 cells^38^. Correlations between the methylome and bacterial abundance from CRC patient biopsies have revealed associations of *F. nucleatum* to hypermethylation of tumor suppressor genes in colorectal cancer^39,40^. These changes promote high microsatellite instability (MSI-H) and high CpG island methylation (CIMP-H)^41,42^. In cancers such as esophageal cancer, it was found that *F. nucleatum* presence correlates with global hypomethylation^43^. These observations establish a key link between epigenomic alterations such as DNA methylation and the tumor microbiome^44,45^. One study profiled intra-tumor bacteria in 29 patients with CRC and obtained mutational signatures of altered genes within the tumor. The authors discovered that *Fusobacterium* was associated with several mutated genes that were involved in cell cycle related pathways^46^. However, it is not clear if the original tumor’s mutational signature enabled *Fusobacterium* invasion or if after invasion, the bacterium and its metabolites promoted genomic instability thereby resulting in increased mutations at these genetic loci. Single-cell RNA-seq of *F. nucleatum* infected HCT116 and HT29 CRC cell lines under normoxia revealed several differentially regulated signaling pathways within 3 h upon infection with a high multiplicity of infection (MOI)^5^. It is not known how long this acute response is sustained as the cells continue to grow and proliferate with intracellular *F. nucleatum* and if lower MOIs elicit a similar response.

The impact of TME factors such as hypoxia on the host transcriptome and epigenome in response to *F. nucleatum* infection has not been systematically investigated. Although it has been documented that *F. nucleatum* enrichment in CRC derived tumor tissue correlates with worse prognosis, this effect is compounded based on the consensus molecular subtype (CMS) of the tumor with mesenchymal subtypes exhibiting a pronounced response^47–49^. In addition, cause and effect are impossible to dissect from clinical samples and it is not clear if measured differences in oxygen levels lead to increased infection or vice versa. These observations underscore the need to explore the impact of host-pathogen interactions in cancer from a holistic perspective within physiologically relevant TME conditions such as hypoxia using a controlled infection protocol to identify direct regulation by infection^50^. Our study used a multiomic approach that combines RNA-seq and H3K27ac ChIP-seq to determine the response to infection of a CRC cell line, HCT116, with *F. nucleatum* in normoxic and hypoxic environments to reveal the sustained influence of infection on host epigenome and transcriptome.

## Results

### *F. nucleatum* infects hypoxia-conditioned tumor cells at an increased rate and elicits increased IL-8 and CXCL1 secretion compared to infection of normoxia-conditioned cells

**Fig. 1:**
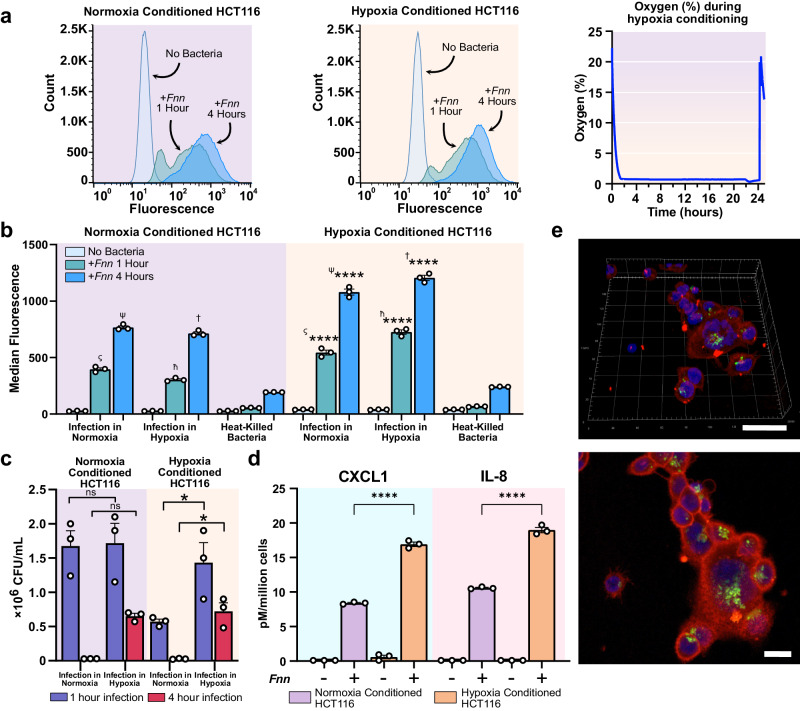
*F. nucleatum* infects hypoxia conditioned cells at an increased rate and elicits increased cytokine secretion in comparison to infection of normoxia conditioned cells. **a** Flow cytometry analysis of infection of normoxic and hypoxic conditioned cells infected with *Fnn* for 1 and 4 h, and a plot of the measured oxygen concentration during hypoxia conditioning. **b** Plot of median fluorescence obtained from flow cytometry using fluorescently stained *Fnn* (FM-1-43) indicates significantly increased infection of hypoxic cells in normoxia and hypoxia compared infection of normoxic cells in normoxia and hypoxic conditions. **c** *Fnn* cell counts in hypoxia at 1 and 4 h are greater than cell counts in normoxia indicating an increased proportion of proliferating bacteria in hypoxia. **d** Cytokine secretions normalized to cell counts (pM/million cells) of IL-8 and CXCL1 are significantly increased upon *Fnn* infection of hypoxic cells compared to normoxic cells at 4 h after infection. **e** Immunofluorescence confocal microscopy of *Fnn* infected HCT116 cells 24 h after initial infection period reveals persistence of intracellular *Fnn*. (Staining: Host membrane red: Membrite 568, Host nucleus blue: NucBlue, *Fnn* green: FM 1-43FX). Scale bar 20μm. Statistical analysis was performed using one-way ANOVA followed by Šidák’s multiple comparisons test (ns: not significant, *p < 0.05, ****p < 0.0001). Symbols (ς, ψ, ћ, †) denote the matched pairwise comparisons made using Šidák’s multiple comparisons test. Source data are provided in Supplementary Data 1.

To investigate whether a hypoxic microenvironment impacts *F. nucleatum* binding and invasion into host cancer cells, we first conditioned HCT116 cells to hypoxia (1% oxygen) and normoxia (18% oxygen) for 24 h and subsequently infected them with *Fusobacterium nucleatum* subsp. *nucleatum* ATCC 23726 (hereafter referred to as *Fnn*) for 1 and 4 h in hypoxia or normoxia. These cells were seeded in normoxia overnight for cell adherence prior to their conditioning to varied oxygen levels. Flow cytometry using fluorescently labeled *Fnn* was used to determine the fractional levels of infection and revealed an increased rate of *Fnn* infection in hypoxia conditioned cells infected under both hypoxia or normoxia in comparison to infection of normoxia conditioned cells in the same conditions (Figs. 1a, b). The infected cells were plated to determine the number of intracellular bacteria 1 h and 4 h after infection and we observed that *Fnn* within hypoxia conditioned cells had increased colony forming units (CFU/mL) 4 h after infection in comparison to normoxia conditioned cells (Fig. 1c). Since we had previously identified IL-8 and CXCL1 to be specifically secreted by HCT116 cells upon *Fnn* infection^19^, we quantified their secretion in response to infection of hypoxia and normoxia conditioned tumor cells. Normalized to cell counts, *Fnn* infection of hypoxia conditioned cells showed a ~two-fold increase in IL-8 and CXCL1 secretion when compared to normoxia conditioned cells (Fig. 1d). Using live immunofluorescence staining and live confocal imaging, we further confirmed bacterial intracellular persistence within the host cancer cells 24 h after the initial 4 h infection (Fig. 1e, Supplementary Fig. 1).

### Hypoxia leads to global changes in the epigenome and transcriptome of HCT116 cells

**Fig. 2:**
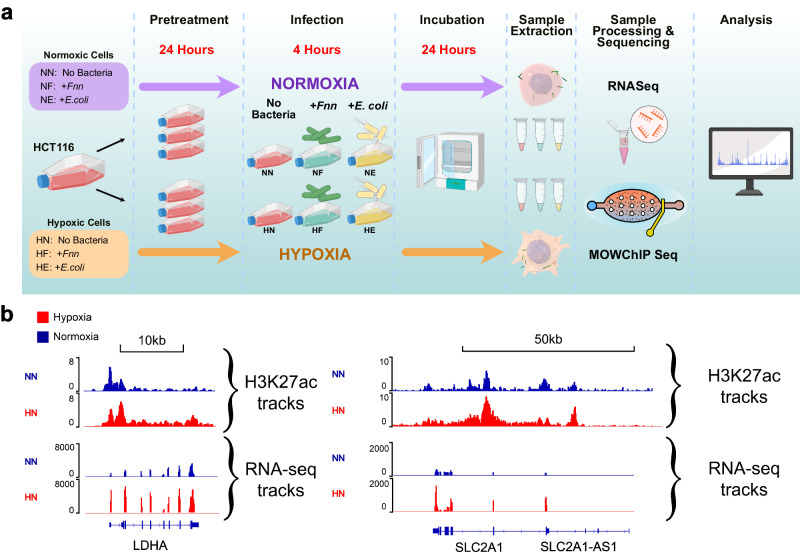
Hypoxia conditioning impacts dynamics of *F. nucleatum* and *E. coli* infection of HCT116 cells. **a** Overview of the experimental protocol for hypoxia conditioning, including pretreatment, infection, continued incubation, sample extraction, and processing. Icons adapted from BioRender.com and schematic designed in Affinity Designer. **b** ChIP-seq tracks and RNA-seq tracks for selected markers of hypoxia: *LDHA* and *SLC2A1* loci for normoxia and hypoxia conditioned samples.

We next sought to quantify epigenomic and transcriptomic alterations of host cancer cells in response to infection in low oxygen. We collected H3K27ac and RNA-seq data of HCT116 cells conditioned to hypoxic and normoxic environments and upon infection with *Fnn* (Supplementary Tables 1, 2). HCT116 cells were first split to two sets of flasks and allowed to adhere to their flasks in normoxia. One set of cells then continued to proliferate in normoxia (18% oxygen) while the other half was exposed to hypoxia (1% oxygen) for 24 h. The time period of 24 h was selected to capture stable and persistent changes in histone modifications and steady-state levels of mRNA transcription^51,52^. After the initial exposure to the oxygen environment, the conditioned cells were infected with *Fnn* for 4 h in their respective oxygen environments in pre-equilibrated media. Infection with non-invasive *E. coli* with otherwise identical conditions was used as a control to validate host cell response. In order to begin to define stable and sustained responses to infection, the cells were further incubated for 24 h in their respective oxygen environments with pre-equilibrated media before they were extracted for analysis. The six datasets that are described in this text are labeled as follows: (a) Normoxia—No Bacteria (NN), (b) Normoxia—infection with *Fnn* (NF), (c) Normoxia—infection with *E. coli* (NE), (d) Hypoxia—No Bacteria (HN), (e) Hypoxia—infection with *Fnn* (HF), and (f) Hypoxia—infection with *E. coli* (HE) (Fig. 2a).

MOWChIP-seq^53^ was applied to obtain genome-wide H3K27ac profiles and an average Pearson correlation coefficient of 0.963 was found between ChIP-seq replicates, which indicated high data quality (Supplementary Fig. 2a). We compared the overall H3K27ac signal at all peak regions (Supplementary Fig. 2b) and found that H3K27ac signals of hypoxia conditioned samples (HN, HE, HF) were found to be significantly higher (*P* < 0.0001) than those of their corresponding normoxia conditioned samples (NN, NE, NF).

RNA-seq data was then analyzed to probe the impact of hypoxia on the host cell transcriptome. We found that the average Pearson correlation coefficient between replicates was 0.925, which indicated high reproducibility of the data (Supplementary Fig. 2c). Consistent with the H3K27ac data (which is an activating mark), we observed significantly increased RNA reads (*P* < 0.0001) in hypoxia conditioned samples with infection (Supplementary Fig. 2d). Extensive genome-wide changes in H3K27ac signal and gene expression were observed in cells cultured in hypoxia compared to normoxia (Fig. 2b). Normalized H3K27ac tracks and RNA-seq tracks were determined for previously reported hypoxia marker genes *LDHA*^54^ and *SLC2A1*^55^. Both genes showed increased H3K27ac signal in hypoxia and the expression levels of the hypoxia marker genes were upregulated in HN compared to NN confirming that the cells were responsive to hypoxia conditioning as expected from prior studies.

### HCT116 cells differential enhancer response to *F. nucleatum* is oxygen dependent

**Fig. 3:**
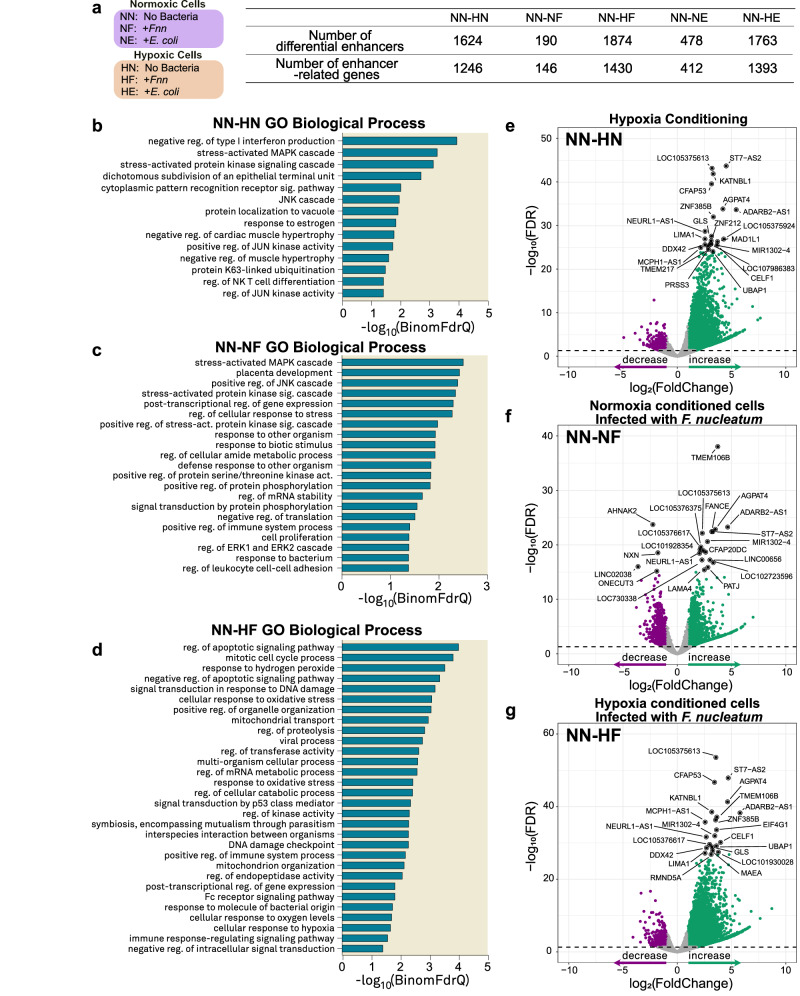
Analysis of differential enhancers reveals *F. nucleatum* infection specific gene regulation based on oxygen environment. **a** Number of genes related to differential enhancers, FDR = 0.05 used as cutoff. **b**–**d** Selected relevant GO terms enriched from NN-HN, NN-NF, and NN-HF specific differential enhancer-related genes. **e**–**g** Volcano plots depicting genes associated with differential enhancers of NN-HN, NN-NF, and NN-HF. The differential enhancers were annotated to their nearest genes. The green points refer to genes linked to enhancers with increased signal compared to NN and purple points refer to those linked to enhancers with decreased signal. Abbreviations: NN Normoxia—No Bacteria, NF Normoxia—infection with *Fnn*, NE Normoxia—infection with *E. coli*, HN Hypoxia—No Bacteria, HF Hypoxia—infection with *Fnn*, and HE Hypoxia—infection with *E. coli*. Source data are provided in Supplementary Data 2.

The histone mark H3K27ac labels active enhancers and modified enhancer activities that are actively involved in CRC progression and relapse^56–58^. Active enhancers are identified as regions with high H3K27ac signal that do not intersect with promoters^59,60^. We examined the number of enhancers under the various conditions tested and performed differential enhancer analysis between normoxia conditioned cells infected by *Fnn* and *E. coli* and normoxia uninfected controls (NN-NF, NN-NE), and hypoxia conditioned cells infected by *Fnn* and *E. coli* versus their normoxia uninfected controls (NN-HF, NN-HE) (Fig. 3a). The number of enhancers generally increased when cells underwent hypoxia treatment (NN = 4008, NF = 5898, NE = 9912, HN = 13081, HF = 11869, HE = 16676). For samples infected with *Fnn*, the number of differential enhancers of the cells infected under hypoxia (NN-HF) (1874) was higher than cells infected under normoxia (NN-NF) (190). Similarly, for samples infected with *E. coli*, the number of differential enhancers of the cells infected under hypoxia (NN-HE) (1763) was substantially higher than cells infected under normoxia (NN-NE) (478). These findings indicate that *Fnn* infection under hypoxia resulted in increased epigenomic changes when compared to infection in normoxia conditioned cells.

To determine the unique impacts of *Fnn* infection under hypoxia, we first identified the differential enhancers between normoxia control and hypoxia uninfected sample (NN-HN), normoxia *Fnn*-infected sample (NN-NF), and hypoxia *Fnn-*infected sample (NN-HF). The differential enhancers were then assigned to their nearest genes for Gene Ontology (GO) analysis and we obtained the processes and pathways that differed between normoxia *Fnn*-infected samples (NN-NF) and hypoxia *Fnn*-infected samples (NN-HF).

The biological processes enriched in NN-HN differential enhancers revealed changes that hypoxia conditioning (without infection) has on HCT116 cells. The impacted terms include stress-activated MAPK cascade, protein-kinase signaling cascade, JNK cascade, and JUN kinase activity (Fig. 3b). Hypoxia is known to directly impact epithelial-mesenchymal transition through multiple pathways, one of which is the JNK pathway^61^. MAPK signaling cascades are tied with HIF-1 activation and can impact cellular proliferation and differentiation. Chronic hypoxia is also known to upregulate c-Jun activity via HIF-1α^62^. Thus, the observation of these terms in enriched enhancers validates the expected response of these cells to hypoxia. Next, we compared the impact of *Fnn* infection in normoxia (NN-NF) to its impact in hypoxia (NN-HF). From the terms enriched in NN-NF (Fig. 3c), we discovered that *Fnn* infection in normoxia are similar to the pathways differentially regulated due to hypoxia conditioning, including stress-activated MAPK cascade and positive regulation of JNK cascade. We also observed terms such as regulation of ERK1 and ERK2 cascade which is involved in cellular proliferation. The host cell exhibits differential regulation in pathways that recognize the bacterial infection, including response to other organisms, response to biotic stimulus, and response to bacterium. Interestingly, we observe the GO term placenta development in these cells. It is known that *Fnn* colonizes the placenta in a Fap2-dependent manner and can impact stress-related pathways which can result in preterm birth complications^63^. In contrast, we observed more biological processes affected during *Fnn* infection in hypoxia (NN-HF) compared to the impact of hypoxia alone (NN-HN) on the host cells. In hypoxia (NN-HF) (Fig. 3d), we discovered that *Fnn* infection significantly impacts the following biological processes: apoptotic signaling, mitotic cell cycle process, signal transduction in response to DNA damage, mitochondrial transport, cellular response to oxidative stress, signal transduction by p53 class mediator, and symbiosis, encompassing mutualism through parasitism. GO terms enriched during *E. coli* infection in normoxia involved the recognition of infection including interspecies interaction between organisms, viral process, and multi-organism cellular process (Supplementary Fig. 3). We also observed the process, regulation of small GTPase mediated signal transduction, which some strains of *E. coli* are known to activate^64^. In hypoxia, *E. coli* infection predominantly activates biological processes related to immune activation which could indicate activation of host cell defenses in response to infection.

Volcano plots (Fig. 3e–g) revealed genes associated with differential enhancers in NN-HN, NN-NF and NN-HF. We further identified several genes that may play critical roles in the regulation of cellular activities upon infection under normoxia (NN-NF) or hypoxia (NN-HF). Under normoxia, we found that infection with *Fnn* modulated the expression of the following genes: *CBX7, LINC010189, SEMA6B, MYO1C, CARD8, ELN*, and *TMOD3*. In hypoxia, these include *WDYHV1, TSPAN19, INFGR2, CLUAP1*, and *SND1*. These genes have been previously found to adversely impact CRC progression. For example, CARD8 (caspase recruitment domain) participates within inflammasomes which recruit Caspase-1 and is involved in pattern recognition and signals for the degradation of disordered and misfolded proteins^65^. A homolog of CARD8, CARD3, has previously been shown to increase migration of *Fnn* infected cells by activating autophagy signaling in response to bacterial peptidoglycan^66^.

### Transcriptomic analysis reveals differential gene expression in hypoxic and normoxic cells upon *F. nucleatum* infection

**Fig. 4:**
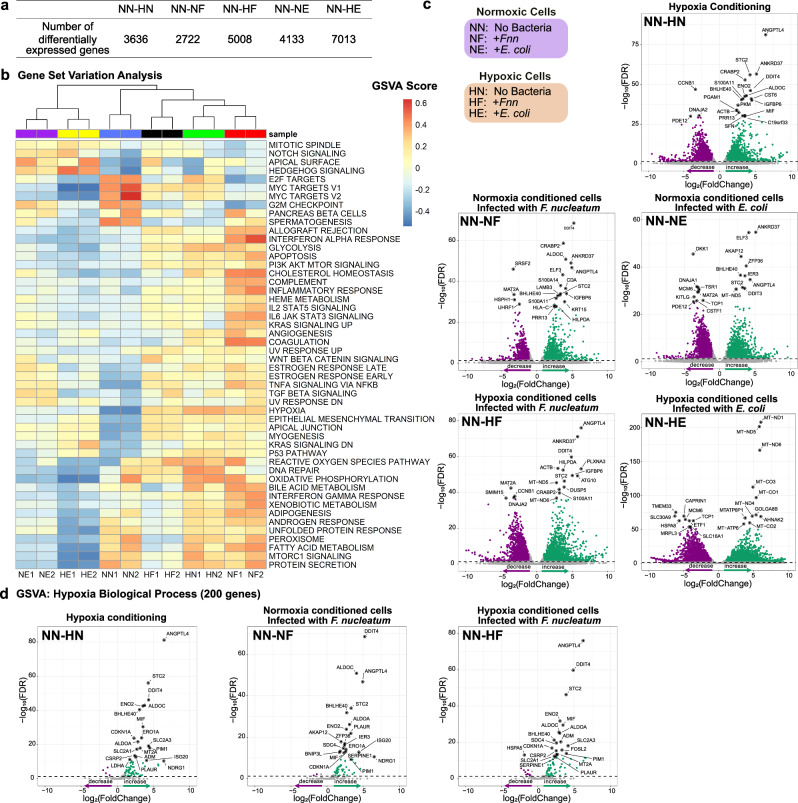
Transcriptomic analysis reveals differential gene expression in hypoxic and normoxic cells upon *F. nucleatum* infection. **a** Number of differentially expressed genes, FDR = 0.05 used as cutoff. **b** Gene set variation analysis (GSVA) of *Fnn* and *E.coli* infection of HCT116 cells in normoxia and hypoxia. **c** Volcano plots showing genes associated with differential expressed genes of NN-HN, NN-NF, NN-HF, NN-NE, and NN-HE. **d** Volcano plots showing DEGs specifically associated with the hypoxia pathway in GSVA for NN-HN, NN-NF, and NN-HF. The green points refer to genes with upregulation compared to NN and purple points refer to those with downregulation. Abbreviations: NN Normoxia - No Bacteria, NF Normoxia—infection with *Fnn*, NE Normoxia—infection with *E. coli*, HN Hypoxia—No Bacteria, HF Hypoxia—infection with *Fnn*, and HE Hypoxia—infection with *E. coli*. Source data are provided in Supplementary Data 3.

Using transcriptomic analysis, we identified differentially expressed genes (DEGs) in the six culture conditions tested. For *Fnn* infected samples, the number of DEGs of cells infected under hypoxia (NN-HF) (5008) was greater than that of cells infected under normoxia (NN-NF) (3636). Similarly, for *E. coli* infected samples, the number of DEGs of the cells infected under hypoxia (NN-HE) (7013) was greater than cells infected under normoxia (NN-NE) (4133). These numbers follow a similar trend to that observed for differential enhancers at the epigenomic level (Fig. 4a).

GSVA (Gene Set Variation Analysis)^67^ was performed to identify specific biological pathways impacted by hypoxia and *Fnn* infection. GSVA analysis evaluates the pathway activities of each single sample by transforming the gene expression-sample matrix into a geneset activity(pathway)-sample matrix. This analysis enabled us to compare the activities of pre-selected genesets between different samples. Here we performed GSVA analysis on all datasets using MSigDB hallmark gene sets. To investigate significantly regulated pathways between samples, we performed differential analysis of the pathway enrichment scores between NN-HN, NN-NF, NN-HF, NN-NE, and NN-HE (Fig. 4b and Supplementary Fig. 4). Based on clustering of impacted pathways found in GSVA, we observed that *E. coli* infected samples (NE, HE) clustered independently of *Fnn* infected samples (NF, HF) indicating that the infection from each of the microbes has dissimilar effects on the hallmark pathways. Furthermore, we observed that HN and NF clustered together indicating similarities in their effects on the hallmark pathways. Accordingly, NF and HF are observed further apart indicating that the effects of *Fnn* infection in normoxia nudges the cell towards a hypoxic phenotype to a greater extent than *Fnn* infection in hypoxia where the cell is already in a hypoxic state.

We next compared the impacted hallmark pathways among the conditions. Comparing NN to HN, the pathways upregulated include PI3K AKT MTOR signaling, TNFα signaling via NFκB, Hedgehog signaling, Wnt and β-catenin signaling, p53 pathway, epithelial mesenchymal transition, and gene sets directly related to hypoxia conditioning including hypoxia and reactive oxygen species (ROS). It is well-documented that hypoxia impacts several of these pathways that play crucial roles in cancer progression^68^. Next, we observed that several biological processes upregulated in HN when compared to NN are also upregulated in NF in comparison to NN. *Fnn* infection in normoxia (comparing NF-NN) are associated with upregulation of cholesterol homeostasis, coagulation, IL6 JAK STAT3 signaling, interferon α response, IL2 STAT5 response, Kras signaling, xenobiotic metabolism, and downregulation of MYC2 targets. Changes in the regulation of cholesterol homeostasis could indicate effects on metabolite synthesis that can impact tumorigenesis^69^. Comparing HF to NN, we observed significant downregulation of fatty acid metabolism, peroxisome, protein secretion, mTORC1 signaling, and unfolded protein response. Concurrently, we see activation of pathways such as PI3K AKT MTOR signaling, TGFβ signaling, Hedgehog signaling, p53 pathway, Kras signaling, IL2 STAT4 pathway, and epithelial mesenchymal transition, but to a lower extent in comparison to NF. The lower levels of activation observed in HF may be because hypoxia conditioning on its own has a substantial impact on these pathways, and *Fnn* infection targets the same pathways. Furthermore, hypoxia-related genes are activated upon *Fnn* infection in normoxia indicating that perhaps *Fnn* is able to simulate a favorable hypoxic intracellular niche within normoxic cells by inducing differential regulation of the same gene networks. These sustained responses parallel the acute response observed from GSEA analysis of single cell analysis of *Fnn* infected HCT116 and HT29 cells from prior studies^5^. In contrast, *E. coli* infection in normoxia (NE) mildly activates hypoxia, glycolysis, Hedgehog signaling, TNFα signaling via NFκB, IL2 STAT5 signaling, and Notch signaling. *E. coli* infection in hypoxia (HE) strongly downregulates MYC Targets V1 and V2, mTORC1 signaling, E2F signaling, interferon γ response, and TGFβ signaling.

Volcano plots of all the DEGs (Fig. 4c) were created for NN-HN, NN-NF, NN-HF, NN-NE, and NN-HE conditions. The top 50 upregulated and downregulated genes that exhibited the largest fold change within significance in *Fnn* and *E. coli* infected hypoxic cells are listed in Supplementary Figs. 5–7. We observed notably distinct genes differentially regulated in these two infection conditions and with respect to the two oxygen environments. In addition, we observed differential regulation of miRNAs (microRNAs) and lincRNA (long intergenic non-coding RNA).

Next, we analyzed the expression of genes specifically within the hypoxia pathway from GSVA for uninfected and *Fnn* infected samples. Of the 200 genes within the hypoxia pathway, our data revealed 193 differentially expressed genes (Fig. 4d). Comparing NN-NF to NN-HN, we observed common trends in the upregulation of *ANGPTL4, DDIT4, STC2, ALDOC, BHLHE40, NDRG1, PLAUR*, and *CDKN1A*. These genes have been previously characterized as biomarkers for CRC and have been directly correlated with increased aggressiveness of CRC. For example, *ANGPTL4* (angiopoietin-related protein 4) plays a role in mediating glycolysis and prior studies have shown that it facilitates *Fnn* colonization in CRC^70^. The significant commonalities of overexpression of these key genes in NN-HN and NN-NF indicates that *Fnn* infection strongly regulates the same and related biological pathways impacted due to hypoxia stress.

### Oxygen environment and infection with *F. nucleatum* modulates differential enrichment of transcription factor binding motifs

**Fig. 5:**
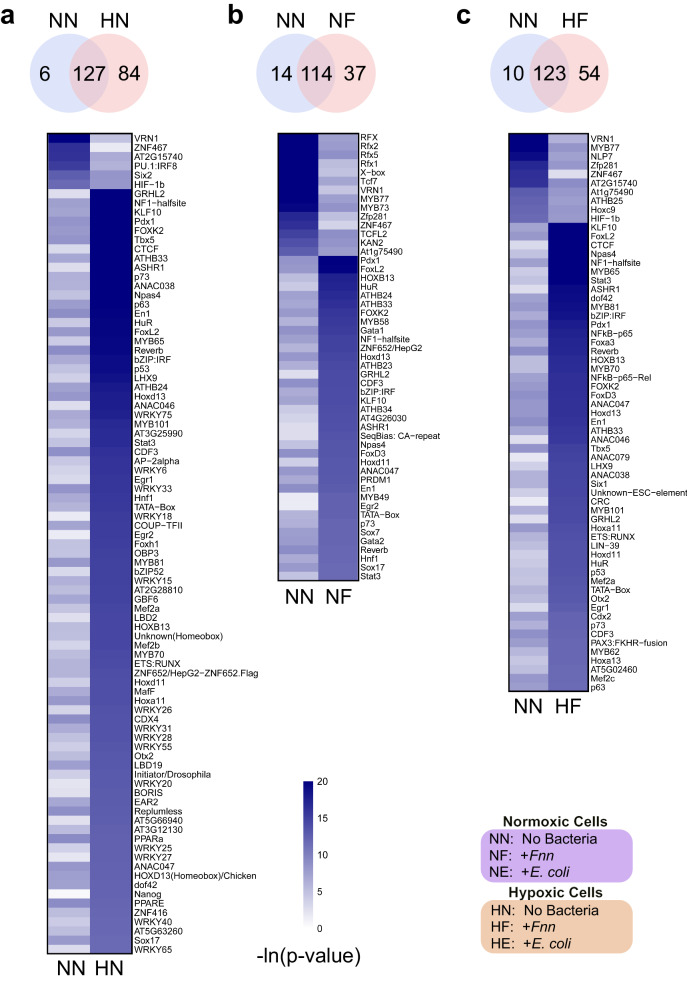
Transcription factor motif analysis in hypoxic and normoxic *F. nucleatum* infected cells reveals oxygen dependent variation in binding. Heatmaps show motifs that are significantly enriched (*p* < 1 × 10^−5^) in one condition but not enriched (*p* > 1 × 10^−4^) in the other. **a** NN and HN. **b** NN and NF. **c** NN and HF. Abbreviations: NN: Normoxia—No Bacteria, NF Normoxia—infection with *Fnn*, HN Hypoxia—No Bacteria, and HF Hypoxia—infection with *Fnn*. Source data are provided in Supplementary Data 4.

To further investigate the changes induced by *Fnn* infection and hypoxia at the transcriptional level, we searched for transcription factor (TF) binding motifs that were significantly enriched under our various treatment conditions^71^. Differentially enriched TFs were identified in NN-HN, NN-NF, and NN-HF (Fig. 5 and Supplementary Table 3). Several TFs associated with CRC were found in NN-NF including P73, STAT3, GATA2, and HUR. To determine *Fnn* specific effects in hypoxia, we analyzed the TFs from NN-HF after removing common terms with NN-HN, and identified differential binding of MYB77, ZFP281, HOXC9, HOXA13, and PAX3:FKHR fusion. These TFs have been implicated in  CRC oncogenesis. The *MYB* family of transcription factors are well-known to promote metastasis in CRC^72^. Studies have shown that HOXA13 (Human class I homeobox A13) is involved in Wnt signaling and β-catenin translocation and promotes colon cancer cell proliferation, migration, and invasion^73^. ZFP281(ZNF281) are zinc finger proteins that have been shown to act as oncogenes promoting growth and metastasis in CRC^74^. In addition, among the enriched TFs in NN-HF, we further identified NFκB-P65, FOXA3, FOXD3, SIX1, CDX2, and MEF2C which have been found to impact tumor progression. For example, SIX1 from the family *sine oculis*, are critical in developmental regulation and the control of cell differentiation. They have been implicated in tumorigenesis, EMT, metastasis, and the Warburg effect in tumor cells^75^. Together, these findings reveal variable transcriptional regulation induced by *Fnn* in high and low oxygen environments that may directly contribute towards tumor progression.

## Discussion

The predominant mechanisms that regulate gene activation and gene repression are histone modifications, DNA methylation, chromatin-associated complexes, non-coding RNAs, and RNA splicing factors, which are collectively referred to as epigenetic reprogramming. Regulation of epigenetic modifications can play an important role in tumor initiation and progression. Invasive bacteria can employ various strategies to passively or actively target these epigenetic mechanisms within host cells which may act to subvert host defense, evade immune signaling, and allow pathogen persistence^76^. The composition of the TME and cellular crosstalk can also play a critical role in the host cell response to infection^77^. Specifically, we hypothesized that tumor hypoxia could be conducive to an anaerobic organism such as *F. nucleatum* to facilitate invasion and intracellular persistence and represents a physiologically relevant niche to study host-microbial interactions. We, therefore, investigated the sustained patho-epigenetic and transcriptomic changes induced upon *F. nucleatum* infection of CRC cells in high and low oxygen environments in order to extract the unique contribution of hypoxia on infection dynamics and host cell response.

There are significant practical challenges to investigate this host-microbial interaction in vivo. The first relates to oxygen measurement. Conventional methods of measuring oxygen levels within tumors are direct approaches which include using needle electrodes (Eppendorf polarographic oxygen electrode), EPR oximetry, and using phthalocyanine-based sensors^78^ or indirect assessments using magnetic resonance imaging (MRI) or PET. These techniques have limited precision, which is essential to investigate direct effects elicited by microbes found at low abundance^79^ and sequestered to microniches^5^ within the tumor. The next challenge is to simultaneously identify specific microbial localization within the tumor before excision. Currently, microbiome research predominantly uses techniques such as 16 S rRNA gene sequencing, immunohistochemistry, and fluorescence in situ hybridization (FISH), which are not suitable for real-time in vivo measurements^4^. Moreover, complex interacting factors within the TME confound individual host cell response to intracellular microbes. Prior studies have approached this challenge by using data from ex vivo samples and correlating hypoxia gene expression with hypoxia exposure and simultaneously assessing microbial abundance^80^. Thus, an in vitro system capable of controlling and modulating these parameters is essential to dissect specific host responses to intracellular microbes.

Using an in vitro co-culture model, we show that hypoxia, a critical TME factor, modulates host tumor cell response to *F. nucleatum* infection. Multiple previous studies have shown the response observed due to infection with *F. nucleatum* is highly dependent upon the genotype and phenotype of the host cell as well as on the specific strain of *Fusobacterium* used^31^. Because of our work in previous studies, we used *F. nucleatum* subsp. *nucleatum* ATCC 23726 (*Fnn*) and a well characterized CRC cell line, HCT116. HCT116 is derived from a MSI positive CRC which occurs in about 12-17% of all CRC^81^. This heterogeneity within tumor subpopulations plays a role in microbial infection where mesenchymal subtypes of CRC are more susceptible to infection than the other subtypes^47,48^. Thus, it is worth noting that these results may not be representative of all CRC, and cell lines derived from alternative subtypes may respond differently to infection and hypoxia^82^. We first demonstrated that *Fnn* infection of HCT116 cells is rapid and elevated in hypoxia conditioned cells in comparison to normoxia conditioned cells. Furthermore, the observed two-fold increase of IL-8 and CXCL1 cytokine secretion upon infection in hypoxia indicates a more robust response of these cells to infection. We have previously characterized the role of these two cytokines in promoting cell migration of HCT116 cells^19^ and hence higher secretion could accelerate the induction of migratory phenotypes within the tumor. In addition, it has been previously shown that hypoxic CRC cells can promote metastasis of neighboring normoxic cells through IL-8/p65 signaling^83^.

Acute cytokine secretion by host cells in response to infection is only one aspect among a multitude of phenotypic and genotypic alterations induced by intracellular infection by *Fnn*. We next characterized the sustained epigenetic imprints and transcriptomic response of the host cell to persistent *Fnn* infection and how hypoxia modulates this response. Our data revealed that 52-h total hypoxia treatment of HCT116 cells increased global H3K27ac, indicating an overall increase in active gene expression. There are limited studies that have previously investigated the impact of hypoxia on H3K27ac levels. Hypoxia can both activate and repress different gene families whose expression is correlated with multiple histone marks^84,85^. Acute hypoxia has been shown to increase HIF-A (hypoxia inducible transcription factor) binding which increases transcriptional activation^51^. It was found that transcriptional output in HCT116 cells upon 90 min of exposure to hypoxia was positively correlated with pre-existing levels of H3K27ac. Another study that subjected HepG2 cells to hypoxia for 4 h demonstrated increased acetylation of H3 in the presence of acetate^86^. Taken together, these findings reveal a complex interconnection between hypoxia and H3K27 acetylation that could vary based on the total duration of hypoxia exposure. Our data supplements these existing findings and reveals increased H3K27ac upon 52-h treatment with hypoxia, indicating activated gene expression at this time point linked with epigenomic reprogramming. This response was observed in all of the hypoxia conditioned samples tested with and without infection.

Using gene ontology (GO) analysis of enriched differential enhancers, we compared the biological processes impacted in the following conditions: NN-HN, NN-NF, and NN-HF. In hypoxia, *Fnn* impacts pathways including apoptotic signaling, signal transduction in response to DNA damage, mitochondrial transport, and signal transduction by p53 class mediator. Previous research has shown that *Fnn* can cause DNA double stranded breaks via the p53 pathway in oral squamous cell carcinoma^87^. Furthermore, aberrant p53 regulation can impact multiple cancer-related pathways including Wnt signaling to impact cell proliferation^88^. Our findings demonstrate the sustained regulation of these pathways over time (24 h) beyond initial infection to impact tumor progression. The impact of *Fnn* infection on mitochondrial transport needs to be explored further and can be related to ROS generation. In fact, there were more biological pathways impacted in NN-HF in comparison to NN-NF and NN-HN, indicating that *F. nucleatum* does play a more active role in hypoxia conditioned cells. Future analysis of affected GO terms will help us understand how these processes directly impact infection dynamics and downstream effects in bacterial-cancer crosstalk.

Using GSVA of the RNA-seq data (Fig. 4 and Supplementary Fig. 4), we determined at a global level, the impacted hallmark pathways of *Fnn* infection in hypoxia. Initial clustering of common pathways revealed that HN and NF clustered together. In normoxia, infection with *Fnn* upregulates several pathways that are also upregulated by hypoxia conditioning alone. These same pathways are further altered during *Fnn* infection of hypoxia conditioned cells. By contrast, *E. coli* infection downregulates several of these pathways in both normoxia and hypoxia and accordingly clustered separately in GSVA (Supplementary Fig. 4). These observations indicate that the host cells respond variably to *Fnn* and *E. coli* infections. To determine impacted pathways due to infection in hypoxia, we conducted pairwise comparisons of NN-NF and NN-HF and observed that hypoxia related genes are upregulated in NN-NF. This indicates that *Fnn* infection impacts genes that are already linked to hypoxia conditioning, potentially foreshadowing a mechanism by which the bacterium modulates its intracellular environment to support its survival. We also observe the downregulation of DNA repair pathways in NF and HF, indicating that intracellular *Fnn* likely enhances the tumor’s susceptibility to acquire new mutations by destabilizing DNA repair mechanisms. Comparing NN-HF, we see infection impacts similar pathways such as TNFα, p53, EMT, Hedgehog signaling, and hypoxia pathway. This again indicates *Fnn* infection acts similar to hypoxia stress on the host cells. Under hypoxia, *Fnn* is able to infect at a greater rate and efficiency, by way of subverting already highly active host signaling. Since hypoxia conditioning drastically impacts malignant transformation, we hypothesize that infection with *Fnn* targets the same pathways to impact malignancy.

In most eukaryotic cells, mitochondria and peroxisomes primarily help in maintaining the redox balance within cells. In addition to phagocytosis and intracellular detoxification, peroxisomes play an important role in oxidative metabolism. Our observations of the downregulation of peroxisome and mTORC1 signaling in *Fnn* infection in hypoxia could indicate that *Fnn* could actively avoid its killing and phagocytosis by targeting and downregulating signaling pathways related to peroxisome. Other intracellular bacteria have harnessed the peroxisome’s function in maintaining cellular redox equilibrium for their survival^89^. Peroxisomes are also hubs for fatty acid metabolism, and hence we observe concurrent downregulation of fatty acid metabolism in HF. The observed lower activation of stress related pathways in HF compared to HN, likely help *Fnn* avoid killing related to oxidative stress. Furthermore, it has been identified that the peroxisome is essential for mediating innate immune response to microbial infection^90^, and thus its downregulation could also be a mechanism by which *Fnn* is able to subvert host defenses. These observations need to be explored further.

Another form of post-transcriptional control involves regulation by miRNAs (microRNAs) and lincRNA (long intergenic non-coding RNA). Our data reveals the selective regulation of miRNAs (*MIR6504*), and lincRNAs (*LINC01089*, *LINC00173*, *LINC02343, LINC01589, LINC02101, LINC02352, LINC01564, LINC02321, LINC02273, LINC01011*) upon *F. nucleatum* infection, whose roles have not been previously characterized in CRC. miRNAs regulate post-transcriptional gene repression by binding to the 3’ UTR of mRNA molecules causing repressed translation and encouraging degradation of the target mRNA. lincRNAs can regulate protein gene expression by acting as a scaffold of histone modifying complexes, binding to transcription factors, or their DNA targets, and direct binding to RNA polymerase or sequestering miRNAs. Several miRNAs have been shown to be downregulated in CRC. In a CRC xenograft model, it has been shown that *F. nucleatum* causes resistance to oxaliplatin and 5-fluorouracil via the downregulation of miR-4802 and miR-18a*^16^. Inhibitors to these two miRNAs caused increased drug resistance and transfection with the miRNAs reversed *F. nucleatum* induced CRC chemoresistance.

Interestingly, we did not observe increased expression of ICAM, which has previously been shown to impact HCT116 adhesion to HUVECs and extravasation^21^. However, this expression could be stimulated based on cell-cell interactions with the endothelium which we do not test in our experiments. In addition, we observed *F. nucleatum* induced downregulation of METTL3, which has been previously shown to mediate metastasis via m6A epi-transcriptional modification^91^. Furthermore, the top upregulated and downregulated gene categories upon *F. nucleatum* infection contained several cancer-related genes that could be uniquely activated upon *F. nucleatum* cancer cell binding (Supplementary Figs. 5–7).

Other histone modifications, post-transcriptional and post-translational modifications, intracellular and extracellular proteomic profiles, splicing variants, and protein trafficking can contribute to the complexity of these interactions to manifest differential outcomes within cancerous tissue. Furthermore, we must account for the phenotypic variability of the cells in their signaling responses. Technologies with increased sensitivity like MOWChIP (sensitivity of <100 cells)^60^ as used in this study can be harnessed to explore the impact of *F. nucleatum* infection present in low cell abundance within clinical samples^79^. We acknowledge the limitation of the current study using a single cell line representing a subset of colorectal cancers. This study aimed to primarily dissect the unique impact of hypoxia on infection dynamics of *F. nucleatum* on host cells. We further acknowledge that normoxia adapted cell lines can differ in their transcriptomic and epigenomic profiles from their in vivo counterparts and our in vitro model reveals host-microbial interactions by amplifying signaling pathways which is infeasible in vivo^92^. Further validation of our findings will be enhanced by using 3D spheroid models, organoid models, and other preclinical models. Additionally, it will be essential to identify what factors in non-cancerous cell lines preclude *Fnn* infection and its impact on cancer-related pathways to determine possibilities for therapeutic intervention.

**Fig. 6:**
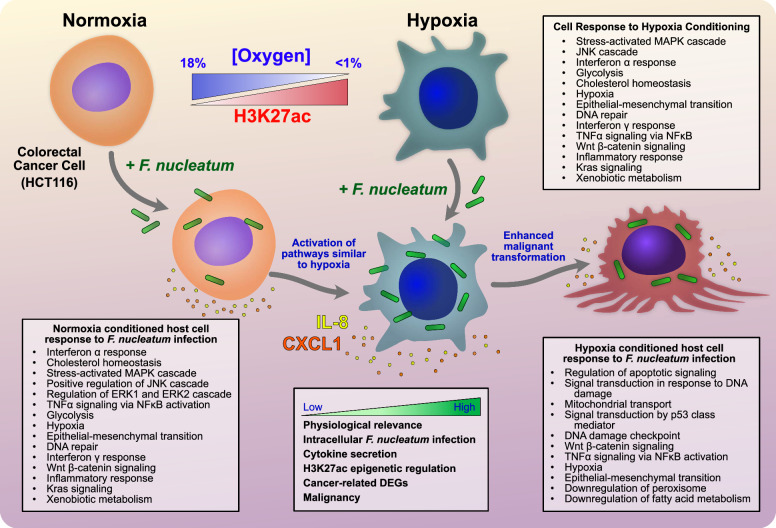
Summary schematic depicting *F. nucleatum* intracellular infection and subsequent host HCT116 colorectal cancer (CRC) cell response in normoxia is similar to hypoxia conditioning and amplifies malignant transformation. Schematic and cell representation created and designed in Affinity Designer.

In conclusion, our data suggests that HCT116 host cell phenotypes modulated by the TME governs its response to *F. nucleatum* infection. Specifically, we report that hypoxia conditioning of host cells primes them for enhanced *F. nucleatum* infection (Fig. 6). Our data further revealed that *F. nucleatum* can modulate its intracellular environment by promoting differential gene expression akin to hypoxia conditioning, which in turn could create an anaerobic niche that contributes to its intracellular persistence. As hypoxia is known to induce aggressive cancer phenotypes, our observations of *F. nucleatum* inducing its own hypoxic niche could explain why infected tumors result in poorer patient outcomes when compared to tumors void of this bacterium. Our results suggest that sustained epigenetic reprogramming of multiple cancer-associated pathways continues to support *F. nucleatum* persistence and prolongs stimulation of tumor progression. In summary, the impact of the tumor microbiome on cancer progression has coalesced two previously disparate fields of study (cancer biology and infectious disease), providing new opportunities to interrogate host-microbial interactions from a holistic perspective and harness this knowledge to develop new cancer therapies.

## Materials and methods

### Bacterial cell culture

*Fusobacterium nucleatum* subsp. *nucleatum* ATCC 23726 was grown under anaerobic conditions^19^. Frozen cultures of bacteria were streaked on solid agar plates made with Columbia broth (Gibco) substituted with hemin (5 μg/mL) and menadione (0.5 μg/mL) (CBHK) and grown in an anaerobic chamber (90% N_2_, 10% H_2_, and 10% CO_2_) at 37 °C (Whitley A35 anaerobic workstation). Single colonies were then retrieved and inoculated in liquid cultures of CBHK media and grown for ~16 h anaerobically to reach a mid-exponential growth phase. The optical density at 600 nm was measured to be 0.5 (Implen OD600 DiluPhotometer) and the bacteria were resuspended in PBS and used for the experiments.

*Escherichia coli* TOP 10 was grown in Luria Broth (LB) in a shaker incubated at 37 °C overnight. Bacterial culture was diluted to an optical density (600 nm) of 0.5 before being used for experiments.

### Epithelial cell culture

HCT116 cells were purchased from ATCC (ATCC CCL-247) and grown in McCoys 5A media supplemented with 10% FBS and 1% Penicillin and Streptomycin. Cells were grown in a normoxia incubator at 37 °C with 5% CO_2_. Cells were passaged with gentle trypsinization (0.25% trypsin with EDTA) and re-seeding at 1:10 ratio. Cells used in the experiments did not exceed passage 12. Cells were re-seeded in 12-well plates or T25 flasks for experiments. For epigenomic and transcriptomic experiments, cells were first seeded at a 1:12 ratio in T-25 flasks and allowed to adhere for 12 h in normoxia. After adherence, one set of cells continued to grow in normoxic conditions and another set was transferred to a hypoxic incubator for pre-treatment.

### Hypoxia incubator

The hypoxic environment was generated using a custom-built hypoxic chamber in the form of a modified incubator (ThermoFisher, 3578), an oxygen profiling microsensor (PreSens, PM-PSt7) connected to an oxygen meter (PreSens, OXY-1 ST), and a gas flow controller supplying 100% nitrogen gas, controlled using a custom Python code and Arduino circuit. The chamber reduced oxygen levels to <1% within an hour of initiation and desired oxygen levels were maintained through the course of the experiment. PreSens oxygen sensor calibrations were routinely run per manufacturer’s instructions.

### Flow cytometry and cell sorting

Bacteria were first stained with FM 1-43FX lipophilic styryl dye (Invitrogen F35355) (5 μg/mL) for 5 min to stain the outer membrane of the bacteria. The stained cells were spun down at 1000 g for 3 min, washed with media and resuspended in its original volume to be used for experiments. Normoxic and hypoxic pre-treated epithelial cells were infected with stained *Fnn* at 50:1 multiplicity of infection (MOI, Bacteria:Epithelial) for 1 or 4 h in their respective oxygen environments. Following infection, cells were washed twice with PBS, trypsinized, and collected for flow cytometry and cell sorting experiments. Cells were then loaded into an S3e flow cytometer (Bio-rad) and gated for single cells. 50,000 cells per sample were analyzed for green fluorescence due to intracellular *F. nucleatum*. Median fluorescence was determined using FlowJo10 before transferring data to GraphPad Prism for statistical analysis.

### Immunofluorescence imaging

Live immunofluorescence visualization was achieved by staining epithelial cells according to manufacturer’s instructions with NucBlue (ThermoFisher Scientific 451 R37605) and Membrite 568/580 (Biotium BTM30095) for the cell’s nucleus and membrane respectively. *F. nucleatum* was stained with FM 1-43FX lipophilic styryl dye (Invitrogen F35355) (5 μg/mL) for 5 min to stain the outer membrane of the bacteria. Adhered epithelial cells were infected with *F. nucleatum* at 50:1 MOI for 4 h. Z-stack images were then taken with an LSM800 confocal microscope (Zeiss) to confirm intracellular invasion of bacteria. 60 slices within a total z height of 22 µm were obtained at a resolution of 1024 × 1024 pixels upon excitation with lasers 488 nm, 561 nm, and 405 nm. To confirm persistence of intracellular bacteria, z-stack images were again taken at 24 h and 3D structures were recreated using Zen Blue.

### Chemokine quantitation

HCT116 cells were infected with *F. nucleatum* in normoxia and hypoxia after pre-treatment for 4 h. The supernatant was collected and filtered using a Spin-X column to remove cell debris and bacteria. ELISA was used to quantify cytokines IL-8 and CXCL1 using Duo Kits (R&D Systems DY208, DY275). The ELISA plate was prepared by coating with Capture antibody overnight and was blocked with the reagent diluent the following day for 1 h. Samples and standards were added to the plate and incubated at room temperature for 2 h. The plate was then washed thrice with a Washing Buffer and the detection antibody was added. The plate was incubated for another 2 h. The plate was again washed thrice and incubated with Streptavidin-HRP for 30 min. The plate was washed once again before adding the Substrate solution and the color change was monitored for 20 min. The reaction was stopped using the Stop solution and the absorbance at 450 nm was collected using a spectrophotometer.

### Chromatin preparation

~22,000 HCT116 cells were used in each condition to produce two replicates of ChIP-seq libraries (~10,000 cells per library) and one input library (~2,000 cells). Cell culture medium was removed by centrifugation at 300 g for 5 min at 4 °C. Cells were then washed twice with 1 mL cold PBS and suspended in 60 μL PBS supplemented with 0.6 μL PIC and 0.6 μL PMSF solution. 60 μL of 2X lysis buffer (4% (vol/vol) Triton X-100, 100 mM Tris-HCl [pH 8.0], 100 mM NaCl and 30 mM MgCl_2_ in Milli-Q water) was added to the tube. The mixture was mixed well and incubated at room temperature for 10 min. Next, 6 μL of 100 mM CaCl_2_ solution and 12 μL of 10 U/μL MNase (in PBS) was added to the tube and vortexed for 5 s. This mixture was incubated at room temperature for 10 min. 13.2 μL of 0.5 M EDTA was then added to the tube to quench the MNase digestion. The mixture was incubated on ice for 10 min, followed by centrifugation at 16,100 g for 10 min at 4 °C. Cellular debris was precipitated to the bottom of the tube and 68 μL of supernatant containing the fragmented chromatin was collected for each ChIP-seq sample. 14 μL of the supernatant was collected for the input sample.

### Beads preparation

~5 μL of protein A-coated Dynabeads (Life Technologies) were transferred to a microcentrifuge tube and washed twice with IP buffer (20 mM Tris-HCl [pH 8.0], 140 mM NaCl, 1 mM EDTA, 0.5 mM EGTA, 0.1% (wt/vol) sodium deoxycholate, 0.1% (wt/vol) SDS, 1% (vol/vol) Triton X-100 in Milli-Q water). The beads were resuspended in 150 μL of IP buffer with 0.5 μg of H3K27ac antibody (Active Motif Inc, cat: 39133), then rotated overnight at 4 °C. The beads were washed with IP buffer three times and resuspended in 5 μL of IP buffer and placed on ice until their use in one ChIP-seq assay.

### MOWChIP-seq

MNase-digested chromatin samples of ~10,000 cells per library were used in MOWChIP-seq to profile H3K27ac. We prepared two ChIP-seq libraries and one input library for each condition. The protocol of MOWChIP-seq was detailed in previous publication^53^. ChIP DNA bound to the beads was collected by phenol-chloroform extraction. The Accel-NGS 2 S Plus DNA Library Kit (Swift Biosciences, Cat# 21024) was used to prepare sequencing libraries. Libraries were sequenced with Illumina HiSeq 4000 with single-end 50 nt read length.

### RNA-seq

Each RNA-seq library was prepared using 25,000 cells. We prepared two technical replicates for each condition. Total RNA was extracted using the RNeasy Mini Kit (74104, Qiagen) and RNase-Free DNase Set (79254, Qiagen). The extracted total RNA was suspended in 30 μL RNase-free water and concentrated by ethanol precipitation. Total RNA was resuspended in 4.6 μL of RNase-free water supplemented with 5% RNase inhibitor (40 U/μL). SMART-seq2 protocol^93^ with minor alterations^59,71^ was then used for reverse transcription to produce cDNA. 2 μL of oligo-dT primer (10 μM), 2 μL of dNTP mix (10 mM) and 4.3 μL of total RNA solution were first mixed well. This mixture (8.3 μL) was denatured at 72 °C for 3 min and immediately stored on ice. Next, 11.7 μL of reverse transcription mix (1 μL of SuperScript II reverse transcriptase (200 U/μL), 0.5 μL of RNase inhibitor (40 U/μl), 4 μL of Superscript II first-strand buffer, 1 μL of DTT (100 mM), 4 μL of 5 M Betaine, 0.12 μL of 1 M MgCl_2_, 0.2 μL of TSO (100 μM), 0.88 μL of nuclease-free water) was added to the 8.3 μl mixture. The total reverse transcription reaction mix was incubated in a thermal cycler at 42 °C for 90 min, followed by 10 cycles of (50 °C for 2 min and 42 °C for 2 min), and finally inactivated at 70 °C for 15 min. The reverse transcription reaction mix was then mixed with 30 μL of PCR mix (25 μL KAPA HiFi HotStart ReadyMix, 0.5 μL IS PCR primers (10 μM), 2.5 μL Evagreen dye, and 2 μL nuclease-free water) and amplified with the program: 98 °C for 1 min, 9–11 cycles of (98 °C for 15 s, 67 °C for 30 s, 72 °C for 6 min). Finally, the cDNA was purified using 40 μL of SPRIselect beads and resuspended in ~7 μL of low-EDTA TE buffer.

The RNA-seq library was made using Tn5-tagmentation. Tn5 transposase (14-17 mg/ml) was produced following a published protocol^94^. Tn5 transposase and T5/T7 transposons were assembled to form Tn5 transposome following a published protocol^95^. Briefly, 1.5 μl of 100 μM T5 transposon (IDT) was mixed with 1.5 μl of 100 μM pMENTS (a 5’-phosphorylated 19-bp mosaic end complementary oligonucleotide, IDT), while 1.5 μl of 100 μM T7 transposon (IDT) was mixed with 1.5 μl of 100 μM pMENTS. 3 μl of T5-pMENTS solution and 3 μl of T7-pMENTS solution were separately incubated at 95 °C for 5 min followed by a slow ramp to 25 °C at −0.1 °C/sec. 2.5 μl of T5-pMENTS solution was then mixed with 2.5 μl of Tn5 transposase (14-17 mg/ml), while 2.5 μl of T7-pMENTS solution was mixed with 2.5 μl of Tn5 transposase. T5-Tn5 solution and T7-Tn5 solution were separately incubated at 37 °C for 1 h to form T5-Tn5 transposome and T7-Tn5 transposome, respectively. 4 μl of T5-Tn5 transposome and 4 μl of T7-Tn5 transposome were mixed to form 8 μl of assembled Tn5 transposome before it was mixed with 1 μl cDNA (containing ~500 pg cDNA) and 1 μl reaction buffer (TNP92110, Lucigen). The reaction mixture was incubated at 37 °C for 1 h. 1 μl of 10 × stop solution (TNP92110, Lucigen) was added to the reaction mixture to stop tagmentation. Two rounds of 1 × SPRIselect beads cleanup were performed to purify tagmented cDNA. The tagmented cDNA was then eluted in 10 μl low EDTA TE buffer. 25 μl KAPA HiFi HotStart ReadyMix was activated at 98 °C for 30 sec and then mixed with 9.5 μl tagmented cDNA and 10 μl Nuclease-free water, followed by incubation at 72 °C for 5 min. 1.5 μl of 25 μM P5 primer, 1.5 μl of 25 μM P7 primer and 2.5 μl 20x Evagreen were then added. The following amplification program was used to achieve a 300-400 RFU increase: 98 °C for 30 s; 11-12 cycles of (98 °C for 10 s, 63 °C for 30 s, 72 °C for 30 s) and 72 °C for 1 min. Amplified library was purified with 0.8 × SPRIselect beads and eluted in 8 μl low EDTA TE buffer. Libraries were sequenced with Illumina HiSeq 4000 with single-end 50 nt read length.

### Enhancers analysis

We conducted random subsampling to reduce the ChIP-seq datasets with >25 million reads to 25 million. We predicted enhancer regions by identifying H3K27ac high regions that did not intersect with promoter regions (TSS ± 2000 bp). First, consensus H3K27ac peak sets were generated for each of the experimental conditions (NN, NE, NF, HN, HE, HF) with Diffbind (3.10.1). Peaks were expanded 500 bp on each side around the summit, forming a 1000 bp long peak width. H3K27ac peak regions that intersected with a promoter region were removed and the remaining regions were designated as enhancers. Differential enhancers between any two experimental conditions were identified using the bioconductor DESeq2 package (1.40.2) with Benjamini-Hochberg method (FDR < 0.05). Gene ontology analysis of differential enhancers was performed using GREAT (4.0.4) with default settings. Motif analysis was performed to determine enriched transcription factor binding motifs among the enhancer regions with HOMER (V4.11, with options –size 1000 –mask –p 16 –nomotif).

### RNA-seq analysis

RNA-seq data were quantified using Salmon (1.10.2) against the hg38 transcriptome using a full decoy. Differential gene expression analysis was performed using DESeq2, where genes with a fold change > = 2 and FDR < 0.05 were considered as significantly differentially expressed. Genes were analyzed for gene ontologies with clusterProfiler (4.8.3).

### GSVA analysis

Gene set variation analysis (GSVA)^67^ (1.48.3) was conducted using the GSVA package on MsigDB Hallmark gene sets. GSVA enrichment scores of each condition were calculated. Differential analysis of enrichment scores between NN-HN, NN-NF, NN-HF, NN-NE and NN-HE were performed using the limma R package (3.56.2). The GSVA heatmaps were plotted using the pheatmap package (1.0.12).

### Statistics and reproducibility

Statistical analysis was performed on GraphPad Prism 9 using t-tests and ANOVA followed by Šidák’s multiple comparisons test, with *P*-value significance denoted by ns (not significant) for *P* > 0.05, *P ≤ 0.05, **P ≤ 0.01, ***P ≤ 0.001, and ****P ≤ 0.0001. All samples for analysis were collected in independent triplicates. Plots were created on GraphPad Prism 9 and figures were designed on Affinity Designer. We prepared two replicates of ChIP-seq libraries (with one input library) and two replicates of RNA-seq libraries for each condition.

### Reporting summary

Further information on research design is available in the Nature Portfolio Reporting Summary linked to this article.

### Supplementary information

Supplementary Information

Description of Additional Supplementary Files

Supplementary Data 1

Supplementary Data 2

Supplementary Data 3

Supplementary Data 4

Reporting Summary

## Data Availability

The source data associated with the figures are provided in Supplementary Data 1–4. The ChIP-seq and RNA-seq data were deposited into Gene Expression Omnibus under accession number GSE246616. All other data are available from the corresponding author on reasonable request.
