# Supplementary material for: Fusobacterium nucleatum infection modulates the transcriptome and epigenome of HCT116 colorectal cancer cells in an oxygen-dependent manner
Source: Commun Biol. 2024 May 8;7:551. doi: 10.1038/s42003-024-06201-w (PMC11079022; doi:10.1038/s42003-024-06201-w)
Supplement: Supplementary file 1 — Supplementary Information [file 42003_2024_6201_MOESM1_ESM.pdf]

Supplementary Tables and Figures

Supplementary Table 1. H3K27ac ChIP-seq metadata. Abbreviations: NN: Normoxia - No Bacteria, NF: Normoxia - infection with *Fnn*, NE: Normoxia - infection with *E. coli* , HN: Hypoxia - No Bacteria, HF: Hypoxia - infection with *Fnn*, and HE: Hypoxia - infection with *E. coli*.

Experimental group	Repli-cate	Total reads	Trimmed reads	Aligned reads	% duplicate reads	Peaks	FRip
NN	1	13425098	12270795	11890069	70.9443	17833	7.50%
	2	18071088	17329893	16907358	74.3666	22160	10.03%
NE	1	25000000	24898398	23618097	28.8138	28590	16.99%
	2	14974664	14919732	14246577	39.684	32849	19.35%
NF	1	17655910	17592292	15767488	43.4656	18455	9.54%
	2	25000000	24792337	21937958	42.898	19219	11.97%
HN	1	17293043	17020373	12757427	62.063	37446	22.49%
	2	23185060	22736499	19140035	50.8794	33606	15.72%
HE	1	18319962	18210613	17070244	42.3342	44723	22.93%
	2	25000000	24922168	23309318	35.3334	47621	23.55%
HF	1	25000000	24624744	20366483	50.8853	34301	19.15%
	2	19375219	18943094	15188467	53.3603	29804	17.94%

Supplementary Table 2. RNA-seq metadata. Abbreviations: NN: Normoxia - No Bacteria, NF: Normoxia - infection with *Fnn*, NE: Normoxia - infection with *E. coli* , HN: Hypoxia - No Bacteria, HF: Hypoxia - infection with *Fnn*, and HE: Hypoxia - infection with *E. coli*.

Experimental group	Replicate	Total reads	Aligned reads	% Aligned reads
NN	1	9002005	8920009	99.09
	2	13399894	13282658	99.13
NE	1	14727006	14529738	98.66
	2	15474426	15254993	98.58
NF	1	13549539	13408193	98.96
	2	17936962	17761651	99.02
HN	1	13668761	13495696	98.73
	2	15085986	14883882	98.66
HE	1	14751367	14240998	96.54
	2	16620283	16008288	96.32
HF	1	14961090	14794994	98.89
	2	10913784	10783782	98.81

Supplementary Table 3. Differentially regulated transcription factors in *Fnn* infection of HCT116 cells subjected to hypoxia conditioning. Abbreviations: NN: Normoxia - No Bacteria, NF: Normoxia - infection with *Fnn*, HN: Hypoxia - No Bacteria, and HF: Hypoxia - infection with *Fnn*.

NN-HN-specific	NN-HF-specific	Common to NN-HN and NN-HF
PU.1:IRF8	MYB77	VRN1
Six2	NLP7	ZNF467
ATHB24	Zfp281	AT2G15740
WRKY75	At1g75490	HIF-1b
AT3G25990	ATHB25	GRHL2
AP-2alpha	Hoxc9	NF1-halfsite
WRKY6	NFkB-p65	KLF10
WRKY33	Foxa3	Pdx1
Hnf1	NFkB-p65-Rel	FOXK2
WRKY18	FoxD3	Tbx5
COUP-TFII	ANAC079	CTCF
Egr2	Six1	ATHB33
Foxh1	Unknown-ESC-element	ASHR1
OBP3	CRC	p73
bZIP52	LIN-39	ANAC038
WRKY15	Cdx2	Npas4
AT2G28810	PAX3:FKHR-fusion	p63
GBF6	MYB62	En1
LBD2	Hoxa13	HuR
Unknown(Homeobox)	AT5G02460	FoxL2
Mef2b	Mef2c	MYB65
ZNF652/HepG2-ZNF652.Flag		Reverb
MafF		bZIP:IRF
WRKY26		p53
CDX4		LHX9
WRKY31		Hoxd13
WRKY28		ANAC046
WRKY55		MYB101
LBD19		Stat3
Initiator/Drosophila		CDF3
WRKY20		Egr1
BORIS		TATA-Box
EAR2		MYB81
Replumless		Mef2a
AT5G66940		HOXB13
AT3G12130		MYB70
PPARa		ETS:RUNX
WRKY25		Hoxd11
WRKY27		Hoxa11
HOXD13(Homeobox)/Chicken		Otx2
Nanog		ANAC047
PPARE		dof42
ZNF416		
WRKY40		
AT5G63260		
Sox17		
WRKY65		

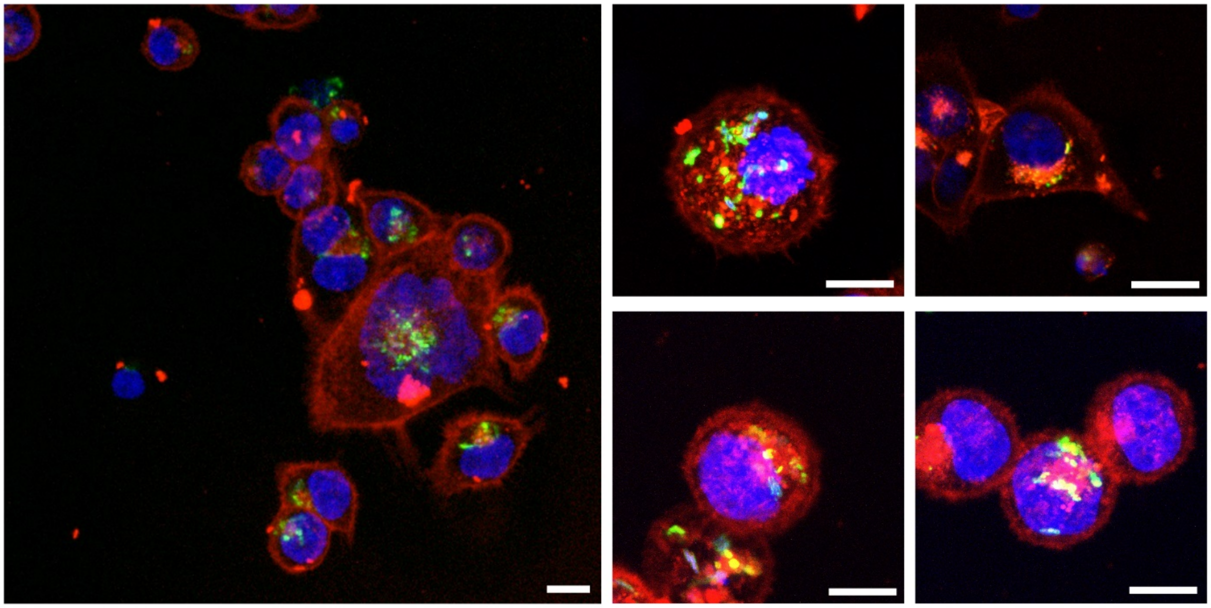

Supplementary Figure 1. Additional images from immunofluorescence confocal microscopy of *Fnn* infected HCT116 cells at 24 hours after initial infection. (Staining: Host membrane red: Membrite 568, Host nucleus blue: NucBlue, *Fnn* green: FM 1-43FX). Scale bar 10μm.

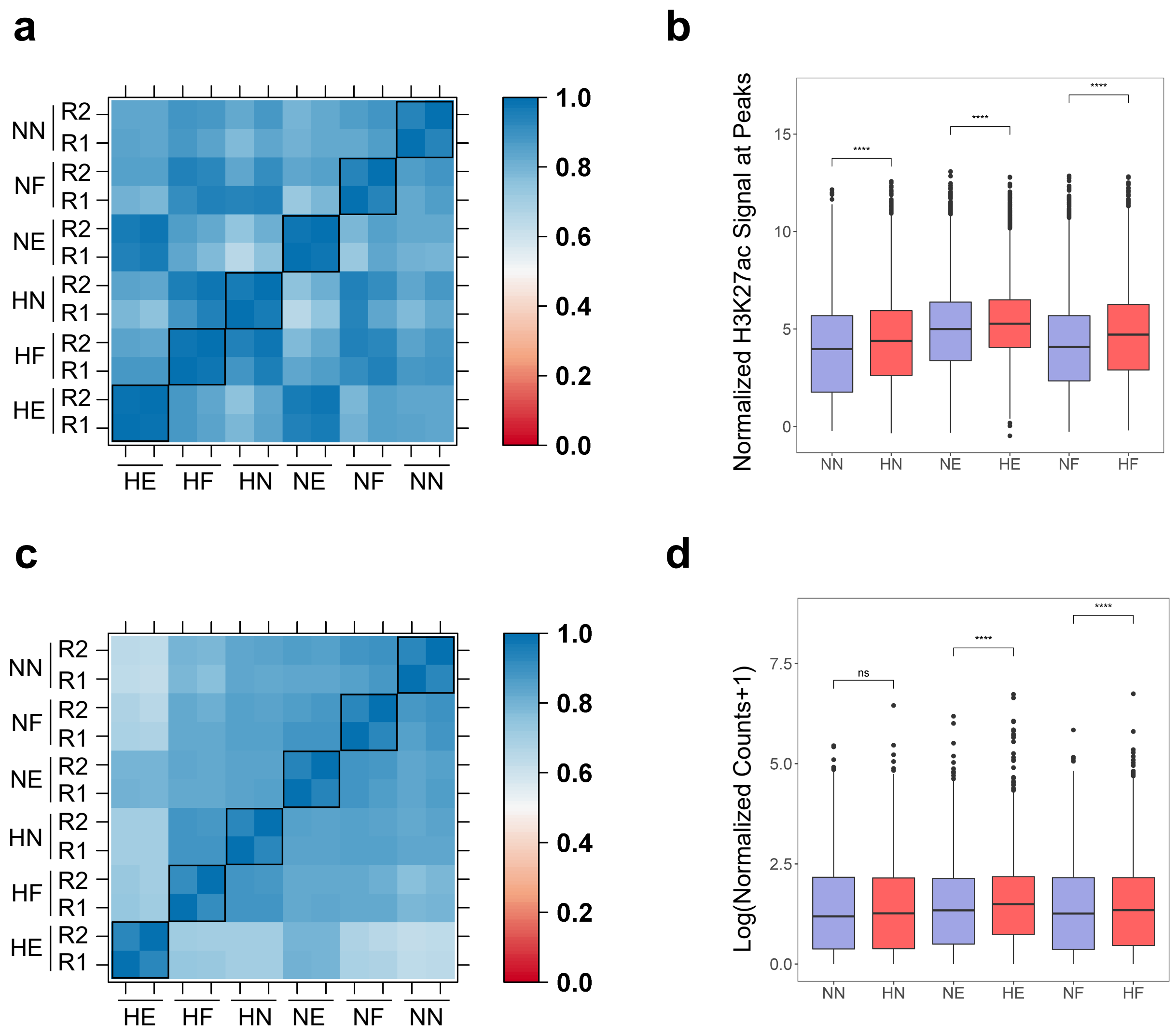

Supplementary Figure 2. H3K27ac and RNA-seq data under various conditions. **a** Pearson correlation of normalized H3K27ac signal around promoter regions (TSS +/- 2 kb). **b** Distribution of normalized H3K27ac signal at peaks. Each data point represents the normalized H3K27ac signal at a peak. The central line of the boxplot represents the median. The upper and lower boundaries of the box delineate the 75th and 25th percentiles, respectively. Each whisker extends to a distance of 1.5 times the interquartile range from the box's edges. **c** Pearson correlation of normalized RNA-seq counts at genes. **d** Boxplots of RNA-seq counts at genes. Each data point represents the normalized read counts at a gene. The central line of the boxplot represents the median. The upper and lower boundaries of the box delineate the 75th and 25th percentiles, respectively. Each whisker extends to a distance of 1.5 times the interquartile range from the box's edges. Abbreviations: NN: Normoxia - No Bacteria, NF: Normoxia - infection with *Fnn*, NE: Normoxia - infection with *E. coli* , HN: Hypoxia - No Bacteria, HF: Hypoxia - infection with *Fnn*, and HE: Hypoxia - infection with *E. coli*. ns (not significant), **** $p<0.0001$.

a

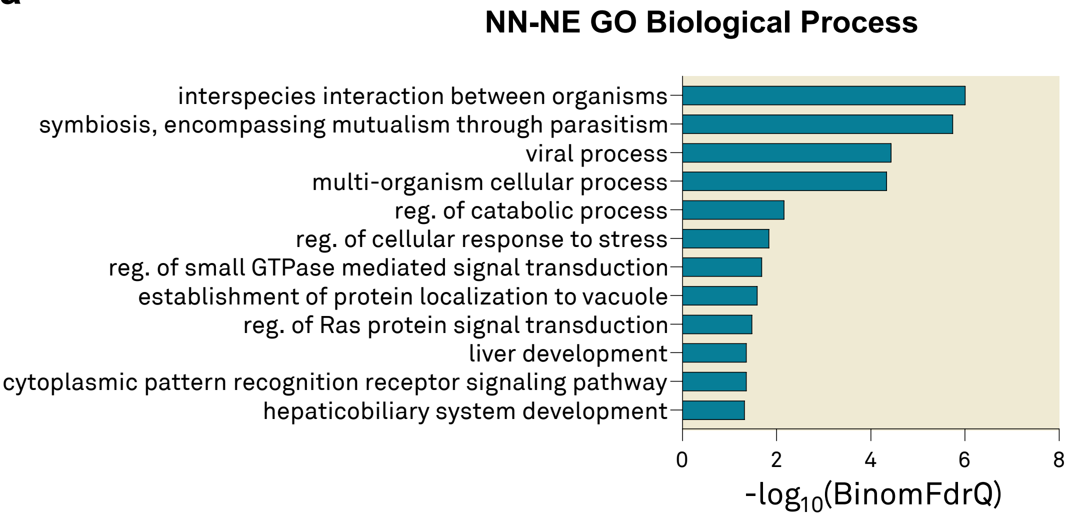

b

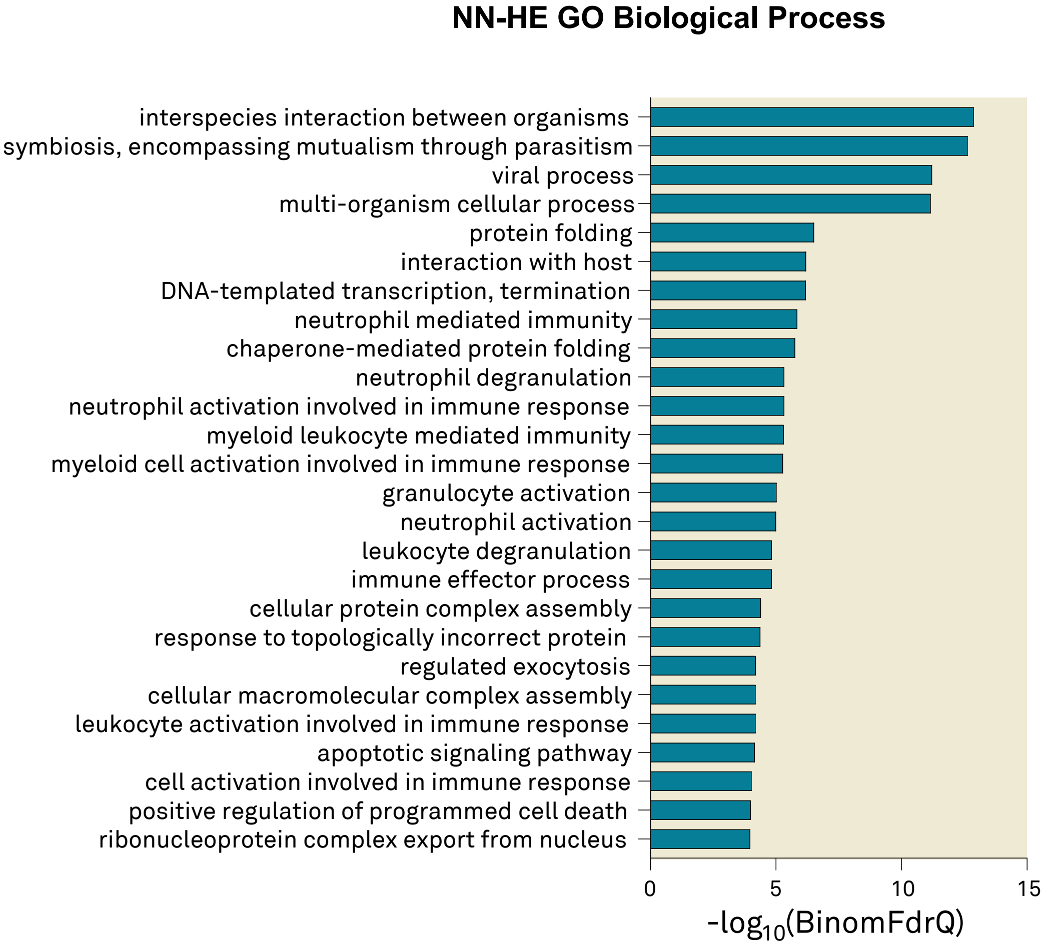

Supplementary Figure 3. Enhancer GO Term enrichment analysis of *E. coli* data. **a** NN-NE. **b** NN-HE. Abbreviations: NN: Normoxia - No Bacteria, NE: Normoxia - infection with *E. coli*, and HE: Hypoxia - infection with *E. coli*.

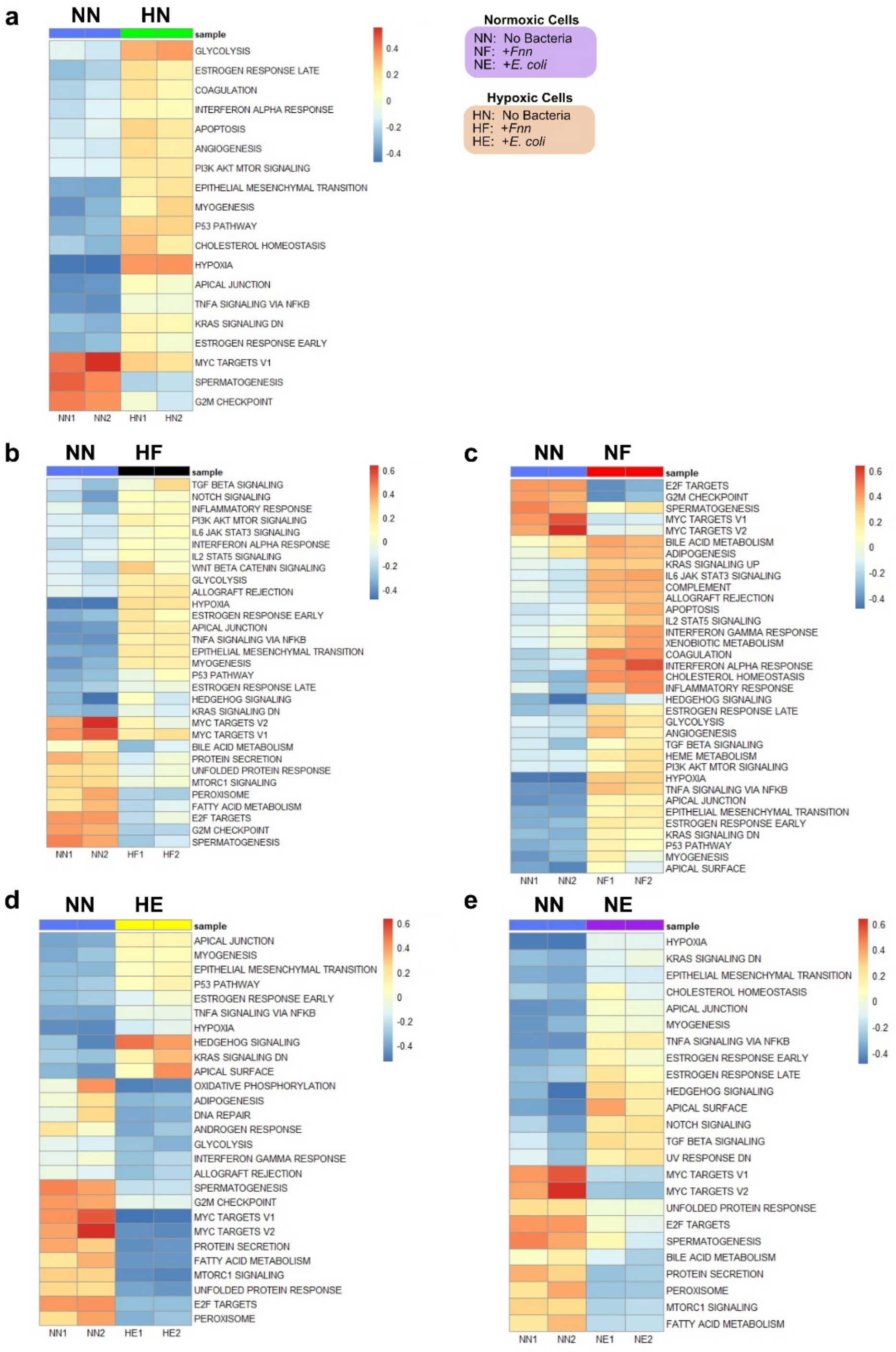

Supplementary Figure 4. Pairwise comparisons of GSVA analysis of RNA-seq data for *Fnn* and *E. coli* infection in normoxia and hypoxia. **a** NN-HN. **b** NN-HF. **c** NN-NF. **d** NN-HE. **e** NN-NE. Abbreviations: NN: Normoxia - No Bacteria, NF: Normoxia - infection with *Fnn*, NE: Normoxia - infection with *E. coli* , HN: Hypoxia - No Bacteria, HF: Hypoxia - infection with *Fnn*, and HE: Hypoxia - infection with *E. coli*.

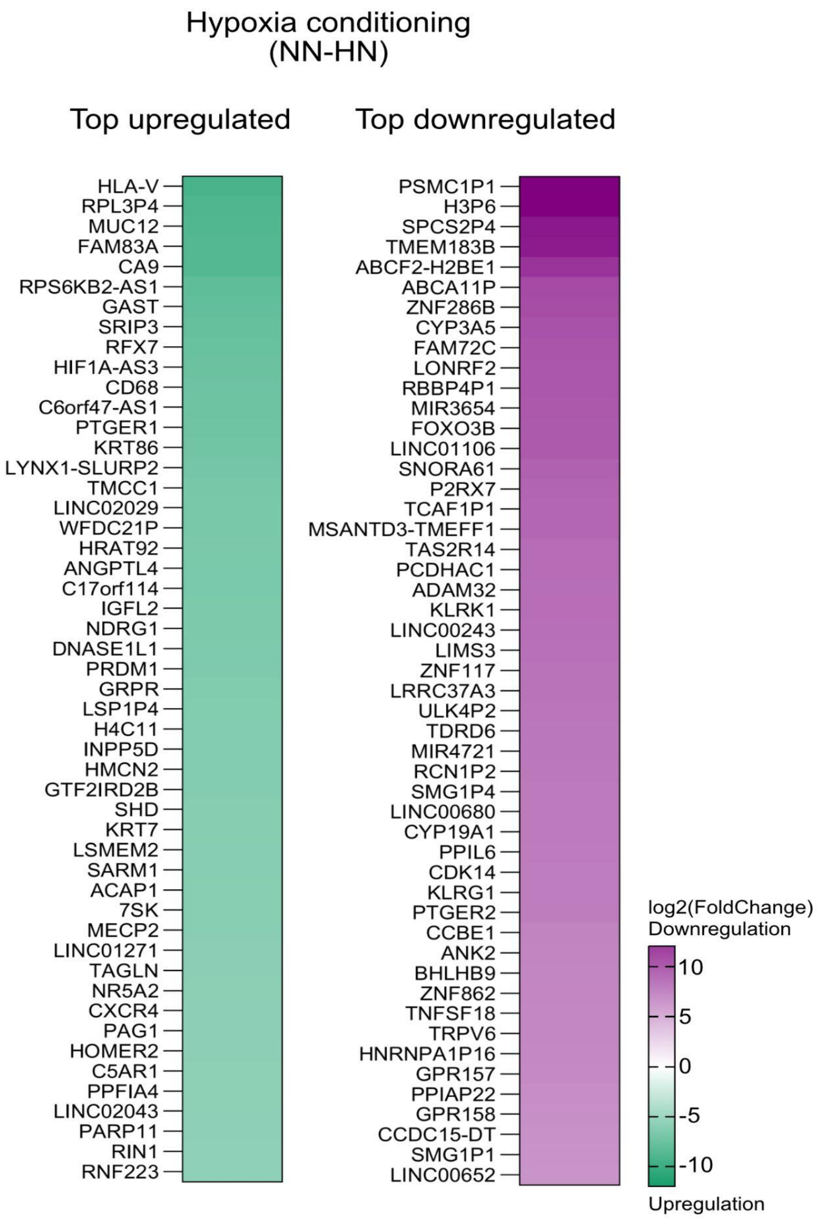

Supplementary Figure 5. Differentially expressed genes in hypoxia conditioned cells. Top 50 upregulated and downregulated DEGs in uninfected hypoxia conditioned cells. Abbreviations: NN: Normoxia - No Bacteria, and HN: Hypoxia - No Bacteria.

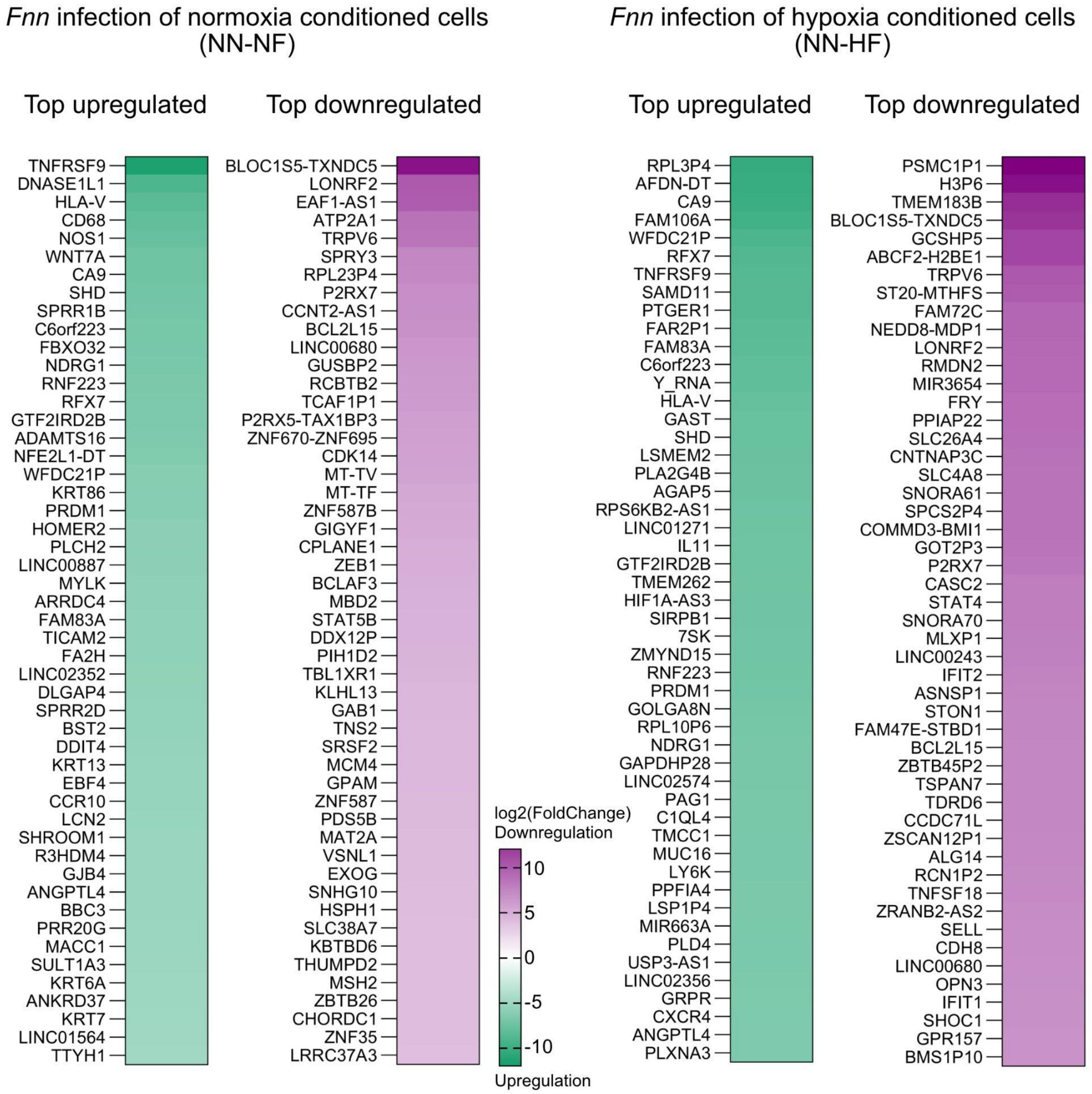

Supplementary Figure 6. Differentially expressed genes in normoxia and hypoxia conditioned cells upon infection with *F. nucleatum*. Top 50 upregulated and downregulated DEGs in normoxia and hypoxia conditioned cells infected with *Fnn.* Abbreviations: NN: Normoxia - No Bacteria, NF: Normoxia - infection with *Fnn*, HN: Hypoxia - No Bacteria, and HF: Hypoxia - infection with *Fnn*.

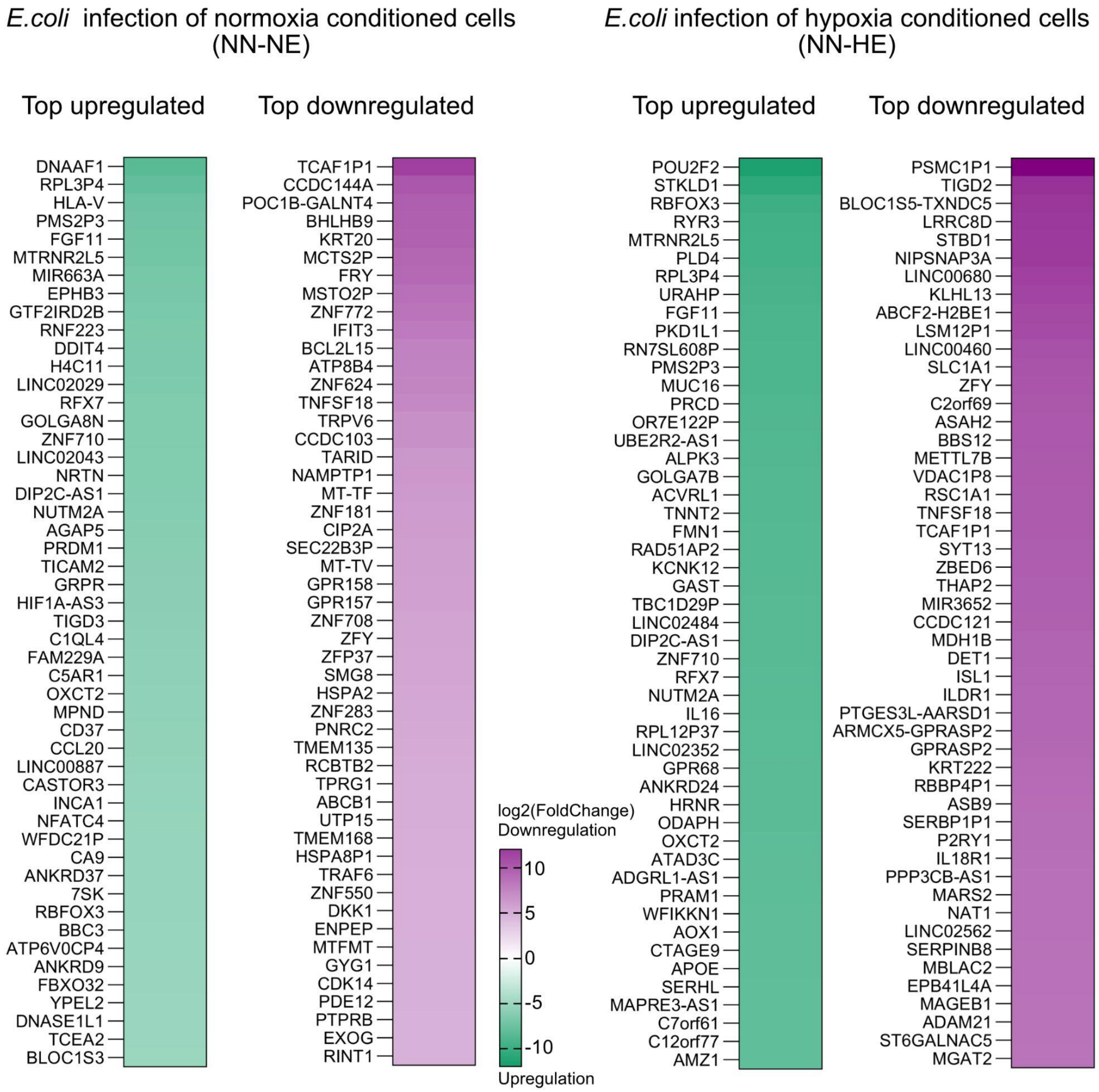

Supplementary Figure 7. Differentially expressed genes in normoxia and hypoxia conditioned cells upon infection with *E. coli*. Top 50 upregulated and downregulated DEGs in normoxia and hypoxia conditioned cells infected with *E. coli.* Abbreviations: NN: Normoxia - No Bacteria, NE: Normoxia - infection with *E. coli* , HN: Hypoxia - No Bacteria, , and HE: Hypoxia - infection with *E. coli*.
