# Supplementary material for: Fusobacterium nucleatum infection modulates the transcriptome and epigenome of HCT116 colorectal cancer cells in an oxygen-dependent manner
Source: Commun Biol. 2024 May 8;7:551. doi: 10.1038/s42003-024-06201-w (PMC11079022; doi:10.1038/s42003-024-06201-w)
Supplement: Supplementary file 2 — Description of Additional Supplementary Files [file 42003_2024_6201_MOESM2_ESM.pdf]

Description of Additional Supplementary Files

File name: Supplementary Data 1
Description: The source data behind the graphs in Figure 1

File name: Supplementary Data 2
Description: The source data behind the graphs in Figure 3

File name: Supplementary Data 3
Description: The source data behind the graphs in Figure 4

File name: Supplementary Data 4
Description: The source data behind the graphs in Figure 5
