# Supplementary material for: Fusobacterium nucleatum infection modulates the transcriptome and epigenome of HCT116 colorectal cancer cells in an oxygen-dependent manner
Source: Commun Biol. 2024 May 8;7:551. doi: 10.1038/s42003-024-06201-w (PMC11079022; doi:10.1038/s42003-024-06201-w)
Supplement: Supplementary file 7 — Reporting Summary [file 42003_2024_6201_MOESM7_ESM.pdf]

nature portfolio

Corresponding author(s): Chang Lu

Last updated by author(s): Mar 26, 2024

Reporting Summary

Nature Portfolio wishes to improve the reproducibility of the work that we publish. This form provides structure for consistency and transparency in reporting. For further information on Nature Portfolio policies, see our Editorial Policies and the Editorial Policy Checklist.

Statistics

For all statistical analyses, confirm that the following items are present in the figure legend, table legend, main text, or Methods section.

n/a	Confirmed	
☐	☒	The exact sample size (*n*) for each experimental group/condition, given as a discrete number and unit of measurement
☐	☒	A statement on whether measurements were taken from distinct samples or whether the same sample was measured repeatedly
☐	☒	The statistical test(s) used AND whether they are one- or two-sided<br>*Only common tests should be described solely by name; describe more complex techniques in the Methods section.*
☒	☐	A description of all covariates tested
☒	☐	A description of any assumptions or corrections, such as tests of normality and adjustment for multiple comparisons
☒	☐	A full description of the statistical parameters including central tendency (e.g. means) or other basic estimates (e.g. regression coefficient) AND variation (e.g. standard deviation) or associated estimates of uncertainty (e.g. confidence intervals)
☐	☒	For null hypothesis testing, the test statistic (e.g. *F*, *t*, *r*) with confidence intervals, effect sizes, degrees of freedom and *P* value noted<br>*Give P values as exact values whenever suitable.*
☒	☐	For Bayesian analysis, information on the choice of priors and Markov chain Monte Carlo settings
☒	☐	For hierarchical and complex designs, identification of the appropriate level for tests and full reporting of outcomes
☒	☐	Estimates of effect sizes (e.g. Cohen's *d*, Pearson's *r*), indicating how they were calculated

Our web collection on statistics for biologists contains articles on many of the points above.

Software and code

Policy information about availability of computer code

Data collection	None
Data analysis	GraphPad Prism 9, FlowJo 10, GSVA on MsigDB Hallmark, limma R package (3.56.2), pheatmap package (1.0.12), Salmon (1.10.2), clusterProfiler (4.8.3), GREAT (4.0.4), bioconductor DESeq2 1.40.2, DiffBind 3.10.1

For manuscripts utilizing custom algorithms or software that are central to the research but not yet described in published literature, software must be made available to editors and reviewers. We strongly encourage code deposition in a community repository (e.g. GitHub). See the Nature Portfolio guidelines for submitting code & software for further information.

Data

Policy information about availability of data

All manuscripts must include a data availability statement. This statement should provide the following information, where applicable:

- Accession codes, unique identifiers, or web links for publicly available datasets
- A description of any restrictions on data availability
- For clinical datasets or third party data, please ensure that the statement adheres to our policy

The ChIP-seq and RNA-seq data were deposited into Gene Expression Omnibus under accession number GSE246616.

Research involving human participants, their data, or biological material

Policy information about studies with human participants or human data. See also policy information about sex, gender (identity/presentation), and sexual orientation and race, ethnicity and racism.

Reporting on sex and gender	n/a
Reporting on race, ethnicity, or other socially relevant groupings	n/a
Population characteristics	n/a
Recruitment	n/a
Ethics oversight	n/a

Note that full information on the approval of the study protocol must also be provided in the manuscript.

Field-specific reporting

Please select the one below that is the best fit for your research. If you are not sure, read the appropriate sections before making your selection.

☒ Life sciences ☐ Behavioural & social sciences ☐ Ecological, evolutionary & environmental sciences

For a reference copy of the document with all sections, see nature.com/documents/nr-reporting-summary-flat.pdf

Life sciences study design

All studies must disclose on these points even when the disclosure is negative.

Sample size	Flow cytometry, bacterial counts, and cytokine analysis were performed using triplicate measurements from distinct experiments and the effect size was large enough to determine statistical significance.
Data exclusions	No data was excluded in the study.
Replication	Multiple runs of the biological experiments showed replication and reproducibility of the results. Two technical replicates of ChIP-seq/RNA-seq datasets were generated for each condition. An average Pearson correlation coefficient of 0.963 was found between ChIP-seq replicates, indicating high quality of the data. For RNA-Seq, the average Pearson correlation coefficient between replicates was 0.925, indicating high reproducibility
Randomization	Samples were collected from a random designated group of flasks or wells as the experiment required. Cells were also acquired from a random selection within the treated groups.
Blinding	Blinding was not performed on this dataset, as the only the relative variation in enhancers, and gene expression is quantified.

Reporting for specific materials, systems and methods

We require information from authors about some types of materials, experimental systems and methods used in many studies. Here, indicate whether each material, system or method listed is relevant to your study. If you are not sure if a list item applies to your research, read the appropriate section before selecting a response.

Materials & experimental systems

n/a	Involved in the study	
☒	☐	Antibodies
☐	☒	Eukaryotic cell lines
☒	☐	Palaeontology and archaeology
☒	☐	Animals and other organisms
☒	☐	Clinical data
☒	☐	Dual use research of concern
☒	☐	Plants

Methods

n/a	Involved in the study	
☐	☒	ChIP-seq
☐	☒	Flow cytometry
☐	☐	MRI-based neuroimaging

Eukaryotic cell lines

Policy information about cell lines and Sex and Gender in Research

Cell line source(s)	HCT116 cells were purchased from ATCC (ATCC CCL-247), Fusobacterium nucleatum subsp. nucleatum ATCC 23726
Authentication	Cell lines were sourced from ATCC
Mycoplasma contamination	Cell line tested negative for mycoplasma contamination
Commonly misidentified lines (See ICLAC register)	n/a

Plants

Seed stocks	n/a
Novel plant genotypes	n/a
Authentication	n/a

ChIP-seq

Data deposition

☒ Confirm that both raw and final processed data have been deposited in a public database such as GEO.

☒ Confirm that you have deposited or provided access to graph files (e.g. BED files) for the called peaks.

Data access links
May remain private before publication.

https://www.ncbi.nlm.nih.gov/geo/query/acc.cgi?acc=GSE246616

Files in database submission

1.NN-H3K27ac-1.fastq.gz
2.NN-H3K27ac-2.fastq.gz
3.NE-H3K27ac-1.fastq.gz
4.NE-H3K27ac-2.fastq.gz
5.NF-H3K27ac-1.fastq.gz
6.NF-H3K27ac-2.fastq.gz
7.HN-H3K27ac-1.fastq.gz
8.HN-H3K27ac-2.fastq.gz
9.HE-H3K27ac-1.fastq.gz
10.HE-H3K27ac-2.fastq.gz
11.HF-H3K27ac-1.fastq.gz
12.HF-H3K27ac-2.fastq.gz
13.NN-input.f.astq.gz
14.NE-input.fastq.gz
15.NF-input.fastq.gz
16.HN-input.fastq.gz
17.HE-input.fastq.gz
18.HF-input.fastq.gz
19.NN-H3K27ac-1.bw
20.NN-H3K27ac-2.bw
21.NE-H3K27ac-1.bw
22.NE-H3K27ac-2.bw
23.NF-H3K27ac-1.bw
24.NF-H3K27ac-2.bw
25.HN-H3K27ac-1.bw
26.HN-H3K27ac-2.bw
27.HE-H3K27ac-1.bw
28.HE-H3K27ac-2.bw
29.HF-H3K27ac-1.bw
30.HF-H3K27ac-2.bw

Genome browser session
(e.g. UCSC)

not available

Methodology

Replicates

2 technical replicates were generated for each ChIP-seq sample. An average Pearson correlation coefficient of 0.963 was found between ChIP-seq replicates

Sequencing depth

We conducted random subsampling to reduce the ChIP-seq datasets with more than 25 million reads to 25 million to facilitate differential analyses. Libraries were sequenced using Illumina HiSeq 4000 with single-end 50 nt read length.

NN-H3K27ac-1 Total reads: 13425098 unique reads: 3900756
NN-H3K27ac-2 Total reads:18071088 unique reads: 4632234
NE-H3K27ac-1 Total reads: 25000000 unique reads: 17796550
NE-H3K27ac-2 Total reads: 14974664 unique reads: 9032118
NF-H3K27ac-1 Total reads: 17655910 unique reads: 9981663
NF-H3K27ac-2 Total reads: 25000000 unique reads:14275500
HN-H3K27ac-1 Total reads: 17293043 unique reads:6560462
HN-H3K27ac-2 Total reads: 23185060 unique reads:11388641
HE-H3K27ac-1 Total reads: 18319962 unique reads:10564353
HE-H3K27ac-2 Total reads: 25000000 unique reads:16166650
HF-H3K27ac-1 Total reads: 25000000 unique reads:12278675
HF-H3K27ac-2 Total reads: 19375219 unique reads:9036544

Antibodies

H3K27ac, Active Motif Inc, cat: 39133

Peak calling parameters

macs2 callpeak -t -c -f BED -g hs -q 0.05

Data quality

NN-H3K27ac-1 peak number:17833
NN-H3K27ac-2 peak number:22160
NE-H3K27ac-1 peak number:28590
NE-H3K27ac-2 peak number:32849
NF-H3K27ac-1 peak number:18455
NF-H3K27ac-2 peak number:19219
HN-H3K27ac-1 peak number:37446
HN-H3K27ac-2 peak number:33606
HE-H3K27ac-1 peak number:44723
HE-H3K27ac-2 peak number:47621
HF-H3K27ac-1 peak number:34301
HF-H3K27ac-2 peak number:29804

Software

1. Trim Galore! 0.6.10
2. fetchChromSizes 332
3. bedtools 2.26.0
4. bowtie2 2.5.0
5. samtools 1.9
6. MACS2 2.2.9.1
7. bedGraphToBigWig 332

Flow Cytometry

Plots

Confirm that:

☒ The axis labels state the marker and fluorochrome used (e.g. CD4-FITC).

☒ The axis scales are clearly visible. Include numbers along axes only for bottom left plot of group (a 'group' is an analysis of identical markers).

☒ All plots are contour plots with outliers or pseudocolor plots.

☒ A numerical value for number of cells or percentage (with statistics) is provided.

Methodology

Sample preparation

Bacteria were first stained with FM 1-43FX lipophilic styryl dye (Invitrogen F35355) (5 µg/mL) for 5 minutes to stain the outer membrane of the bacteria. The stained cells were spun down at 1000g for 3 minutes, washed with media and resuspended in its original volume to be used for experiments.

Normoxic and hypoxic pre-treated epithelial cells were infected with stained Fnn at 50:1 multiplicity of infection (MOI, Bacteria:Epithelial) for 1 or 4 hours in their respective oxygen environments. Following infection, cells were washed twice with PBS, trypsinized, and collected for flow cytometry and cell sorting experiments. Cells were then loaded into a S3e flow cytometer (Bio-rad) and gated for single cells. 50,000 cells per sample were analyzed for green fluorescence due to intracellular F. nucleatum. Median fluorescence was determined using FlowJo10 before transferring data to GraphPad Prism for statistical analysis.

Instrument	Biorad S3e flow cytometry and cell sorter
Software	Biorad ProSort
Cell population abundance	50000 cells per sample was collected and purity was determined by the tightness of the peak and ProSort conditions.
Gating strategy	Since this was a binary experiment that included unstained samples vs stained samples, only the samples with florescently labeled bacteria was detected with the green laser. The experiment was performed at two time points 1 hour and 4 hour and there is a clear demarcation between the no bacteria and plus bacteria sample peaks at 4 hours.

☒ Tick this box to confirm that a figure exemplifying the gating strategy is provided in the Supplementary Information.

Magnetic resonance imaging

Experimental design

Design type	n/a
Design specifications	n/a
Behavioral performance measures	n/a

Acquisition

Imaging type(s)	n/a
Field strength	n/a
Sequence & imaging parameters	n/a
Area of acquisition	n/a

Diffusion MRI ☐ Used ☒ Not used

Preprocessing

Preprocessing software	n/a
Normalization	n/a
Normalization template	n/a
Noise and artifact removal	n/a
Volume censoring	n/a

Statistical modeling & inference

Model type and settings	n/a
Effect(s) tested	n/a

Specify type of analysis: ☐ Whole brain ☐ ROI-based ☐ Both

Statistic type for inference (See Eklund et al. 2016)	n/a
Correction	n/a

Models & analysis

n/a	Involved in the study
☒	☐ Functional and/or effective connectivity
☒	☐ Graph analysis
☒	☐ Multivariate modeling or predictive analysis
